# Genome-wide analysis of MYB transcription factors in four *Rheum* L. plants provides new insights into the synthesis of Anthraquinones

**DOI:** 10.3389/fpls.2025.1558321

**Published:** 2025-05-16

**Authors:** Tao Wang, Shuo Zhao, Bo Wang, Jianan Li, Zengrong Ye, Famei Zhang, Huiyuan Ma, Guoying Zhou

**Affiliations:** ^1^ Key Laboratory of Tibetan Medicine Research, Northwest Institute of Plateau Biology, Chinese Academy of Sciences, Xining, Qinghai, China; ^2^ University of Chinese Academy of Sciences, Beijing, China; ^3^ College of Agriculture and Animal Husbandry, Qinghai University, Xining, China; ^4^ College of Life Sciences, Qinghai Normal University, Xining, China

**Keywords:** MYBs, anthraquinones, genome-wide analysis, RNA-Seq, qPCR, *Rheum* L. plants

## Abstract

*R. tanguticum* (*Rheum tanguticum* Maxim. ex Regel) is a herbaceous plant belonging to Polygonaceae family and *Rheum* L. genus. It holds considerable value in culinary and medicinal realms, primarily due to their rich Anthraquinones (AQs) content. Understanding the molecular mechanisms that regulate AQs biosynthesis is a prerequisite for increasing their yield. MYB transcription factors (TFs) can regulate the synthesis of a variety of plant secondary metabolites. However, only a few research have explored the role of MYB TFs in *Rheum* L. species. In this study, 1054 MYB genes from four *Rheum* L. species were identified. The number of MYB genes in each species was similar, distributed across 11 chromosomes. To investigate the phylogeny of identified MYB TFs, they were classified into four subfamilies. Sequence characteristics, phylogenetic relationships, evolutionary trends, and tissue expression of MYB genes in *Rheum* L. species were further studied. Subsequently, 12 MYB genes were selected, which shown differential expression in different tissues. Further research on these genes indicated a significant correlation with genes in shikimate pathway and polyketide pathway of AQs biosynthesis. Protein-protein interaction simulations in *Arabidopsis thaliana* and qRT-PCR experiments further confirmed this situation. This research lays the foundation for studying molecular mechanisms by which MYB TFs regulates AQs biosynthesis in four *Rheum* L. species.

## Highlights

- A total of 1054 MYB genes were identified in four *Rheum* L. species.
- MYBs reflect the genetic diversity of the three genuine rhubarbs (*R. palmatum*, *R. officinale* and *R. tanguticum*).
- Tandem duplication makes a significant contribution to the expansion of the MYBs in *R. tanguticum*.
- Some MYB genes play a key role in AQs biosynthesis.

## 1 Introduction

*Rheum* L. plants is a tall perennial herb in Polygonaceous family, with about 60 species worldwide, mainly in temperate and subtropical alpine regions of Asia (Wang et al., 2005). Certain species among them possess exceptionally high medicinal and nutritional values, thereby securing a significant position in global applications. *R. palmatum*, *R. officinale* and *R. tanguticum* are *Rheum* L. species included in the Chinese Pharmacopoeia and of significant medicinal value, commonly referred to as “Rhubarbs” (C.P.C., 2020). *R. nobile*, which is similar in habitat and closely related phylogenetically, is utilized only in a restricted geographical area (Tibet). Research on *R. nobile* is currently relatively limited (**Table 1**). Rhubarbs contain a variety of important secondary metabolites, including over 200 types of secondary metabolites (Xiang et al., 2020). Among these active components, AQs such as aloe-emodin, rhein, chrysophanol, etc., exhibit significant pharmacological activity and possess effects such as antibacterial, anti-inflammatory, and anticancer properties (Wang et al., 2021).

**Table 1:** AQs content in four *Rheum* L. species: A comparative review.

Ethnopharmacological classification	Species	Evaluation of *AQs* Content	References
Authentic rhubarbs	*R. tanguticum* *R. palmatum* *R. offcinale*	As most important three species in *Rheum* genus, *R. palmatum* l., *R. tanguticum* Maxim., and *R. officinale* Baill. have been widely used for medicinal (roots).	Su et al., 2020 Tan et al., 2023 Zhuang et al., 2020
Regional rhubarb	*R. nobile*	Besides Authentic rhubarbs, some plants in this genus produced in Hebei provinces et al. also were used as a rhubarb`s substitute by local people.	Yang et al., 2024

AQs and their derivatives are aromatic polyketone compounds that play important roles in plants, such as photoprotection and enhancing plant disease resistance. The complete biosynthetic pathway of anthraquinones in plants remains unclear. However, two incompletely resolved anthraquinone biosynthetic pathways have been tentatively proposed in plants: the polyketide pathway and the shikimate/o-succinylbenzoate pathway. Due to shared intermediates, the MEP (methylerythritol 4-phosphate) and MVA (mevalonic acid) pathways are also involved in the biosynthesis of AQs (Leistner, 1985; Leistner and Zenk, 1968). Therefore, identifying key functional genes involved in the synthesis pathway of AQs compounds is a crucial step in current research to increase the production of AQs in rhubarbs. Recently, some key structural genes involved in AQs biosynthesis have been discovered in some plants, such as polyketide synthase-like enzyme (PKSIII) in *Cassia tora*, and UDP-glycosyltransferase (UGT) in *Fagopyrum esculentum*, etc (Kang et al., 2020; Zhao H. et al., 2023). However, the research identification of transcription factors related to AQs biosynthesis is still almost a blank slate.

Almost all eukaryotic organisms contain the MYB transcription factors (TFs), which are renowned for its ability to regulate a wide range of physiological processes in plants (Martin and Paz-Ares, 1997). These processes include environmental adaptation, hormone signal transduction, development, metabolic regulation, and so on (Allan et al., 2008). The first MYB TF cloned in plants was *ZmMYBC1* in maize (Paz-Ares et al., 1987). MYB TFs is one of the most prominent families of transcription factors in plants, consisting of three conserved functional domains: DNA-binding domain (DBD), transcription activation domain (TAD), and the not yet fully defined negative regulatory domain (NRD). Among them, DBD is the most conserved and commonly referred to as MYB domain (Frampton, 2004). MYB domain is composed of 1-4 repeating units of about 52 amino acids each, forming three alpha-helices, among which the second and third helices form a helix-turn-helix (HTH) structure (Ogata et al., 1996).

Based on adjacent repetitive units, MYB TFs can be divided into 4 subfamilies: 1R-MYB, R2R3-MYB, 3R-MYB, and 4R-MYB (Ogata et al., 1996). In studies identifying and analyzing MYB TFs present in plants, R2R3-MYB TFs are usually the most abundant, followed by 1R-MYB and 3R-MYB TFs. 4R-MYB TFs are less common, and 5R-MYB as well as newly discovered MYB TFs are even rarer (Jin and Martin, 1999; Yanhui et al., 2006; Thiedig et al., 2021; Si et al., 2023). There is a significant variation in the number of MYB genes across different species, ranging from less than 100 to over 500. For instance, 54 MYB genes have been identified and analyzed in mango (Zhang et al., 2022). In contrast, there are 524 in *Gossypium hirsutum* L (Salih et al., 2016). Most studies focus on a single plant species. To date, there is still limited information available on comparative analysis of MYB genes among closely related species.

The available information is scarce in comparison to the significance of MYB TFs and AQs in *Rheum* L. species, particularly for the three types of rhubarbs and their important related species, *R. nobile*. In several influential databases, no research related to MYB TFs of these four *Rheum* L. plants has been found. This study conducted a comprehensive genomic comparative analysis of MYB TFs in four species of *Rheum* L. plants. 263, 267, 259 and 265 MYB genes in *R. tanguticum*, *R. officinale*, *R. palmatum* and *R. nobile* have been achieved respectively. Subsequently, a systematic study was conducted through comparative genomic, HPLC and RNA sequencing (RNA-seq) analyses to explore the phylogenetic differences of MYB TFs among four rhubarbs, focusing on their tissue-specific expression patterns and protein-protein interaction relationships in *R. tanguticum*. The response of MYB genes to multiple confirmed structural genes involved in AQs synthesis provides a basis for further understanding the role of these genes in AQs biosynthesis. These results reflect a comprehensive molecular evolutionary analysis of MYB TFs and a detailed understanding of specific MYB proteins involved in AQs biosynthesis.

## 2 Materials and methods

### 2.1 Data resources

The whole genome sequence and annotation of *R. tanguticum* were obtained from self-sequencing data, while the data for *R. palmatum*, *R. officinale*, and *R. nobile* were sourced from published genomic articles (Feng et al., 2023; Zhang H. et al., 2024; Zhang T. et al., 2024). MYB protein sequences of *Arabidopsis thaliana* were obtained from the TAIR database (https://www.arabidopsis.org/). MYBs Hidden Markov Model (HMM) profile files were downloaded from Pfam database (http://pfam.xfam.org/), including: Myb_DNA-binding (PF00249), Myb_DNA-bind_2 (PF08914), Myb_DNA-bind_3 (PF12776), Myb_DNA-bind_4 (PF13837), Myb_DNA-bind_5 (PF13873), Myb_DNA-bind_6 (PF13921), and Myb_DNA-bind_7 (PF15963).

### 2.2 Identification of MYB genes in four rhubarbs

To identify MYB genes in four *Rheum* L. plants (*R. tanguticum*, *R. palmatum*, *R. officinale*, and *R. nobile*), these strategies were employed. HMM profile for the MYB domain (Pfam accession numbers: PF00249, PF08914, PF12776, PF13837, PF13873, PF13921, and PF15963) was used to identify potential MYB genes in the genomes of four *Rheum* L. species using HMMER 3.4 software (http://hmmer.janelia.org/), with an E-value threshold of 1e-2 (Cui et al., 2024). Only proteins predicted to contain at least one MYB domain by online tool SMART (https://smart.embl.de/) were identified as candidate MYB members in rhubarbs. Subsequently, BLAST analyses (E-value of 1e-10) were conducted using the MYB candidate genes from the four *Rheum* L. plants as queries against the rhubarbs genome to verify the integrity of the identified MYBs from the database of rhubarbs (Altschul et al., 1990; Katiyar et al., 2012; Yao et al., 2024). Sequences obtained from these strategies were merged and redundant sequences were removed. Finally, CDD database (version 3.21) (https://www.ncbi.nlm.nih.gov/Structure/cdd/wrpsb.cgi) were used to further analyze and validate these sequences. Proteins confirmed by this database were considered candidate MYB TFs.

### 2.3 Physicochemical property analysis of four *Rheum* L. plants MYB proteins

Physicochemical properties of MYB TFs, such as molecular weight and isoelectric point, were analyzed using ExPASy (http://www.expasy.ch/tools/pi_tool.html). Protein Parameter Calc in Tbtools (version 2.119) was used to validate and complement these data (Chen et al., 2023).

### 2.4 Construction of phylogenetic tree of rhubarbs MYB TFs

Using the identified 263, 259, 267, 265, 197 MYB TFs from *R. tanguticum*, *R. palmatum*, *R. offcinale*, *R. nobile* and *Arabidopsis thaliana*, two phylogenetic trees were constructed with *R. tanguticum* as the core. Multiple sequence alignments of the conserved MYB domains were conducted using the ClustalW software implemented in MEGA 11 (version 11.0.13) (Kumar et al., 2016). Subsequently, a neighbor-joining (NJ) phylogenetic tree based on the MYB domain alignment was constructed by MEGA 11 according to JTT model (Kumar et al., 2016). Relative branch support was evaluated by 1000 bootstrap replicates, branch lengths were calculated by pairwise comparisons of genetic distances, and missing data were treated by pairwise deletions of gaps. Additionally, the ChiPlot (https://www.chiplot.online/) and iTOL tree (https://itol.embl.de/) websites were used for tree file modification and visualization.

### 2.5 Sequence analysis of MYB TFs in four *Rheum* L. species

Online MEME Suite (https://meme-suite.org/meme/) was used to perform motif analysis on MYB TFs (Bailey et al., 2009). Detected maximum number of motifs was set to 10, with the site distribution set to any, and all other parameters default. The motif identification images were saved. Batch CD-Search tool on the NCBI website (https://www.ncbi.nlm.nih.gov), was utilized to detect conserved domains in each plant species, with the E-value threshold set to 1e-10 and all other parameters default. Additionally, we prepared phylogenetic analysis files and genomic gff3 files for the four *Rheum* L. species.

Based on results from MEME, CD-search, phylogenetic analysis, and the genomic gff3 files, the identified MYB genes from four *Rheum* L. species were submitted to TBtools for protein motif composition and gene structure analysis, with the results visualized in graphical form (Chen et al., 2023).

### 2.6 Analysis of *cis*-acting elements of four *Rheum* L. MYB TFs

2000 bp *cis*-acting elements in the MYB TFs were obtained from the online website Plant CARE (http://bioinformatics.psb.ugent.be/webtools/plantcare/), and redundancies were removed after screening. Chiplot (https://www.chiplot.online/) was used for heatmap visualization.

### 2.7 Analyses of chromosome distribution, duplication events of MYB TFs in four *Rheum* L. plants

Chromosomal distribution information of MYB TFs in four *Rheum* L. plants was derived from the annotated genomic data in their genome database. Gene Location Visualize (Advanced) tool of TBtools was used to visualize the chromosomal localization of MYBs in rhubarbs, and MapChart 2.3.2 was used to validate the results (https://www.wur.nl/). For the syntenic analysis of MYB genes in the four *Rheum* L. species, MCScanX with default settings was used to identify gene pairs of segmental duplications within their genomes (Wang et al., 2012). Tandem duplications were identified as two MYB genes separated by no more than one intervening gene. Ka and Ks of evolution were calculated using the Simple Ka/Ks Calculator (Chen et al., 2023). The divergence times (T) were calculated as T = Ks/(2λ) × 10^-6 Mya, with the approximate value for the clock-like rate λ = 2.44×10^-9 for rhubarbs (Li et al., 2023).

### 2.8 Expression profiling of *R. tanguticum* MYB TFs in various tissues

To determine expression profiles of MYB TFs, we obtained the transcriptomic data of four types of tissues—root, stem, leaf, and seed—from previous studies conducted in our laboratory. Quality control of the raw reads was performed using FastQC (v0.12.0). After trimming Illumina adapter sequences and removing low-quality bases with the FASTX-Toolkit, clean reads were aligned against the reference genome using HISAT2 (v2.2.1) with default parameter settings (Kim et al., 2015; Liu C. et al., 2020). The aligned reads were then converted into Bam files and sorted using SAMtools (v1.9.1). Subsequently, resulting aligned reads were processed by StringTie to assemble reads into genes and measure the expression levels of each gene (Pertea et al., 2015; Liu Y. et al., 2020). The obtained RNA-seq data were standardized using the “limma” package in R software. Subsequently, differentially expressed genes (DEGs) were assessed using the “DESeq2” package. Genes with an adjusted p-value <0.05 and an absolute log2 fold change (FC) >2 across four different tissues were considered as DEGs, with gene expression levels calculated as the average of three biological replicates. After escaping, number of fragments per kilobase of transcript per million mapped reads (FPKM) was used to visualize the distribution of gene expression levels (https://www.bioinformatics.com.cn) (Tang et al., 2023).

Previous studies have identified several genes encoding enzymes involved in the biosynthesis of AQs from the polyketide pathway, including 24 *PKSIII* genes. Candidate genes generated from the initial analysis and structural genes confirmed to be involved in AQs synthesis were characterised by Pearson’s index using OriginPro (2025) to screen for *RtanMYB* TFs that may regulate AQs biosynthesis.

### 2.9 Analysis of the AQs content

The determination of AQs was carried out using a high-performance liquid chromatography (HPLC) system (Agilent 1260 Infinity II). Reference standards for five AQs were obtained from Sigma (USA). Reagents used included methanol, acetonitrile, phosphoric acid, and formic acid (HPLC grade; Shandong Yuwang Group, CHN). A total of 5 AQs were measured, namely, aloe emodin (110795–201007), rhein (110757–200206), emodin (110756–200110), chrysophanol (110758–201013), and physcion (110796–201118).

A conical flask containing 25 mL of methanol was used to weigh in 0.5 grams of powder, and the total weight was meticulously recorded. After 1 hour of heating and refluxing, the sample was cooled to room temperature and reweighed. Any loss of weight during this process was compensated for by the addition of an equivalent volume of methanol. The solution was then filtered, and the resulting filtrate was collected to serve as the final sample solution for the determination of AQs. Chromatographic separation was achieved using a Unitary C18 column (4.6 × 250 mm, 5 μm, 100 Å). The mobile phase consisted of two components: solvent A (0.1% phosphoric acid in water) and solvent B (methanol), with elution performed in ultrapure water. The gradient elution program was as follows: from 0 to 40 min, 85% solvent B and 15% solvent A. Quantification of AQs was performed using the external standard method, with detection at a wavelength of 254 nm and an injection volume of 10 μL. The methodological approach was based on a previous study on rhubarb (Zhao S. et al., 2024) to ensure the accuracy and reliability of the experimental methods.

### 2.10 Subcellular localization

WoLF PSORT (https://wolfpsort.hgc.jp/) was employed to predict the subcellular localization of MYB TFs in four *Rheum* L. plants. DEGs of *R. tangutian* were comprehensively analyzed using WoLF PSORT, TBtool, and Cell-PLoc to determine their locations in subcellular structures.

### 2.11 Protein-protein interaction network of MYB TFs involved in the biosynthesis of AQs in *R. tanguticum*

STRING database with default parameters (https://string-db.org/) was used to predict, execute and visualise the potential protein-protein interaction networks of *RtanMYB* TFs based on known homologs.

### 2.12 Expression verification of candidate MYB TFs in *R. tanguticum*

DEGs related to AQs biosynthesis pathway were verificated by qRT-PCR. Primers were designed using PRIMER 5.0, and primers were validated for specificity using NCBI Primer BLAST, with *R. tanguticum* 18S rRNA as an internal reference (**Supplementary Table S9**). Sample RNA extraction of the four fractions (roots, stems, leaves and seeds) was first performed followed by reverse transcription operations.

Using TB Green Premix Ex Taq II (with TliRNase H Plus) (Takara Biomedical Technology Co., Ltd., Beijing, China) and an ABI 7500 real-time PCR system (Life Technologies, Foster City, CA, USA), transcript levels of these genes were quantified by quantitative real-time PCR (qRT-PCR). qRT-PCR procedures were as follows: denaturation at 95°C for 30 s, followed by 40 cycles of 95°C for 5 s and 60°C for 34 s; and unwinding at 95°C for 5 s, 60°C for 1 min and 95°C for 5 s. The results were obtained by using the ABI 7500 Real-Time PCR System (Life Technologies, Foster City, CA, USA). Data were analysed using the 2^ (-ΔΔCT) method (Schmittgen and Livak, 2008). In this study, each gene was tested using three identical samples, with each set representing an independent biological replicate. Additionally, to ensure the accuracy of the data, we performed three technical replicates for each sample.

## 3 Result

### 3.1 Identification of the MYB genes in the four *Rheum* L. species

1054 MYB TFs were identified from four *Rheum* L. species, including 263, 259, 267, 265 MYB TFs from *R. tanguticum*, *R. palmatum*, *R. offcinale*, *R. nobile* (**Supplementary Table S1**). Based on their relative linear order on chromosomes, they were renamed. Detailed information about these genes could be found in **Supplementary Tables S1–S3**. Identified MYB TFs were classified into 4 types: 1R-MYB, R2R3-MYB, 3R-MYB, 4R and Atypical-MYB (**Supplementary Table S2**). Among these types, R2R3-MYB was most abundant in four *Rheum* L. species. In *R. tanguticum*, *R. palmatum*, and *R. nobile*, the proportions of 3R-MYB and 4R-MYB were relatively small; while in *R. offcinale*, the proportion of 1R-MYB was relatively small. It was worth noting that, unlike general plant species, the number of MYB TFs in the 3R-MYB, 4R & Atypical-MYB groups had expanded to a greater extent in four rhubarbs, especially in *R. tanguticum*; the number of 3R-MYB and 4R & Atypical-MYB TFs (39.55%) was very close to that of R2R3-MYB (45.25%) (**Supplementary Table S2**).

In addition, since four *Rheum* L. species were widespread in the wild and cultivated as economically important crops. Moreover, MYBs played an important role in stress resistance, nutrient acquisition, and metabolic synthesis. Therefore, this study also conducted an internal comparative analysis of 1054 MYB TFs across the four rhubarbs. Results indicated that there was no linear correlation between the number of MYB genes and the size of the genome in each species (**Supplementary Table S2**). Furthermore, the number of MYB TFs varied among them (**Supplementary Table S2**), with significant interspecific divergence among subfamily members, except for the 3R-MYB. The proportion of 1R-MYB in *R. officinale* was relatively low (11.61%) compared to that in *R. nobile* (19.25%). In *R. tanguticum*, the proportion of R2R3-MYB was relatively low (45.25%), but the proportion of atypical MYBs was relatively high (22.43%). Studies had shown that a smaller number of atypical genes (ATGs) was associated with smaller differences between species, suggesting that large number of ATGs tended to undergo extreme forms of birth and death evolution (Nei and Rooney, 2005). MYB family was an important TF family, and its rapid expansion might have enhanced the adaptability of plants to environmental stresses and promoted establishment of survival signal transduction networks, physiology, and metabolic pathways under stress. This pattern was common in the evolution of gene families and was mainly caused by species-specific gene duplication (Panchy et al., 2016).

### 3.2 *Cis*-acting target sequence analysis of genes that encode enzymes involved in AQs biosynthesis reveal the potential role of MYB TFs

Many studies had confirmed the regulatory role of MYB TFs in mediating the biosynthesis of various secondary metabolites. However, there were no reports on the involvement of MYB in AQs biosynthesis. Gene expression regulation was largely mediated by *cis*-regulatory elements (CREs), which played a critical role in modulating gene functions across various biological processes (Lyu et al., 2024). To ascertain whether MYB TFs were involved in the regulation of AQs biosynthesis in rhubarbs, we analyzed the 2-kb upstream promoter regions of 24 type III polyketide synthases (PKSIII) that have been confirmed to participate in AQs biosynthesis in *R. tanguticum*.

45 CREs related to various biological activities were detected, including plant growth and development (**Supplementary Figure S1**). Notably, binding sites for MYB TFs are widely present among these 24 *RtPKSIII* genes. This finding confirmed the potential of MYB TFs to play a regulatory role in AQs biosynthesis.

### 3.3 Phylogenetic analysis of MYB TFs in four *Rheum* L. species

**Figure 1:**
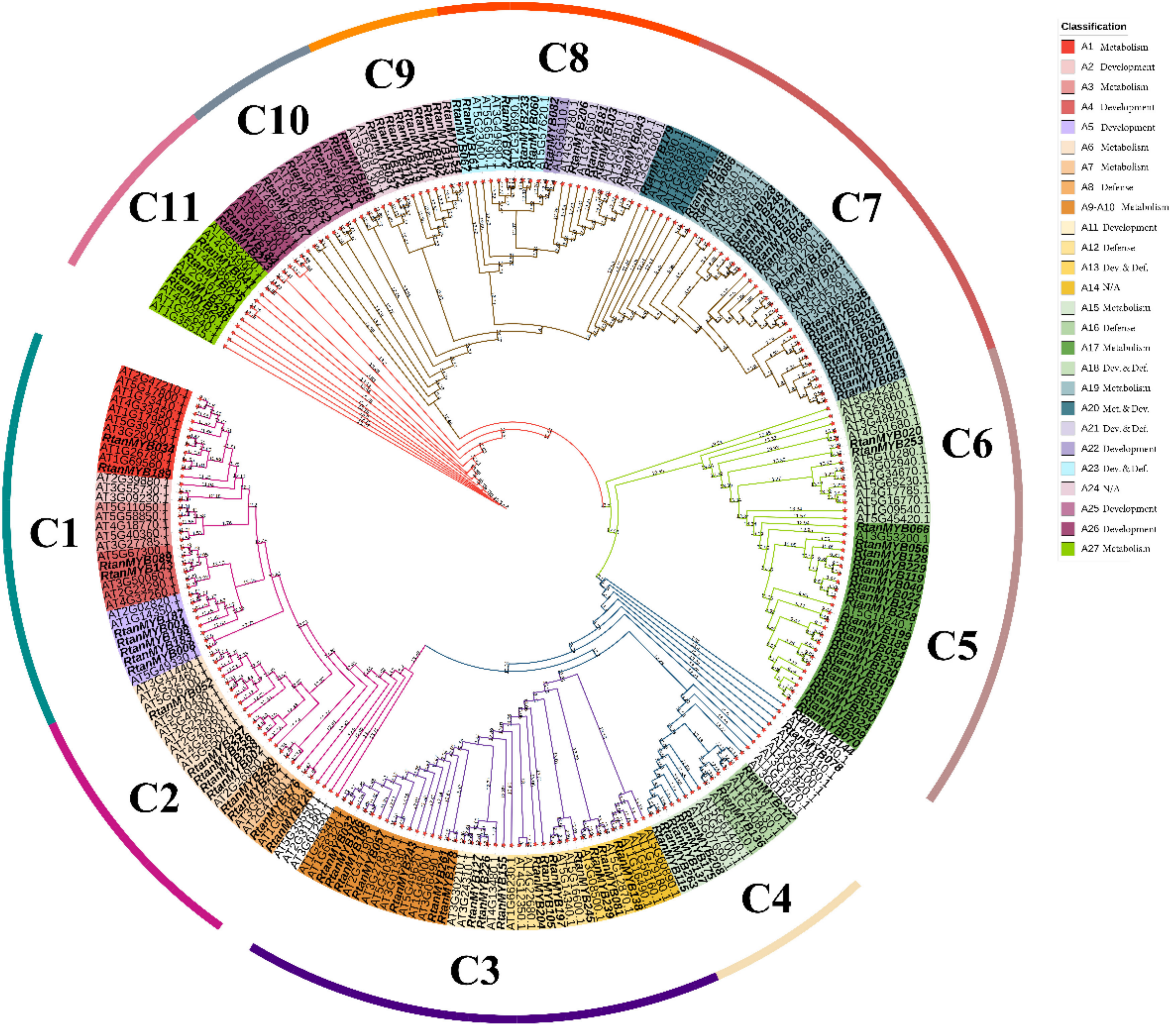
The phylogenetic tree of R2R3-MYB TFs from *R. tanguticum* and *Arabidopsis thaliana*. These proteins were divided into 11 clusters based on their topological structure. Additionally, based on the functional classification of *Arabidopsis thaliana* R2R3-MYBs, 27 subgroups were distinguished, each assigned a number (A1-A27).

In plants, PKSIII genes such as chalcone synthases (CHSs) were involved in the biosynthesis of plant specialized metabolites, especially in the biosynthesis of acetate-pathway-derived flavonoids, stilbenes, and aromatic polyphenols (Lyu et al., 2024; Millard et al., 2019; Kang et al., 2020). CHSs in the flavonoid pathway had been reported to be regulated by R2R3-MYB TFs in many studies (Golovko, 2023; Tuan et al., 2015; Zhu et al., 2023), which provided us with a reliable idea for screening MYB TFs that regulated AQs biosynthesis. Constructing phylogenetic trees based on model plants could offer a promising approach for uncovering gene functions. Therefore, to gain a clearer understanding of the functions of MYB TFs in AQs biosynthesis, phylogenetic trees were constructed using R2R3-MYB TFs from *R. tanguticum* (**Figure 1**).

**Figure 2:**
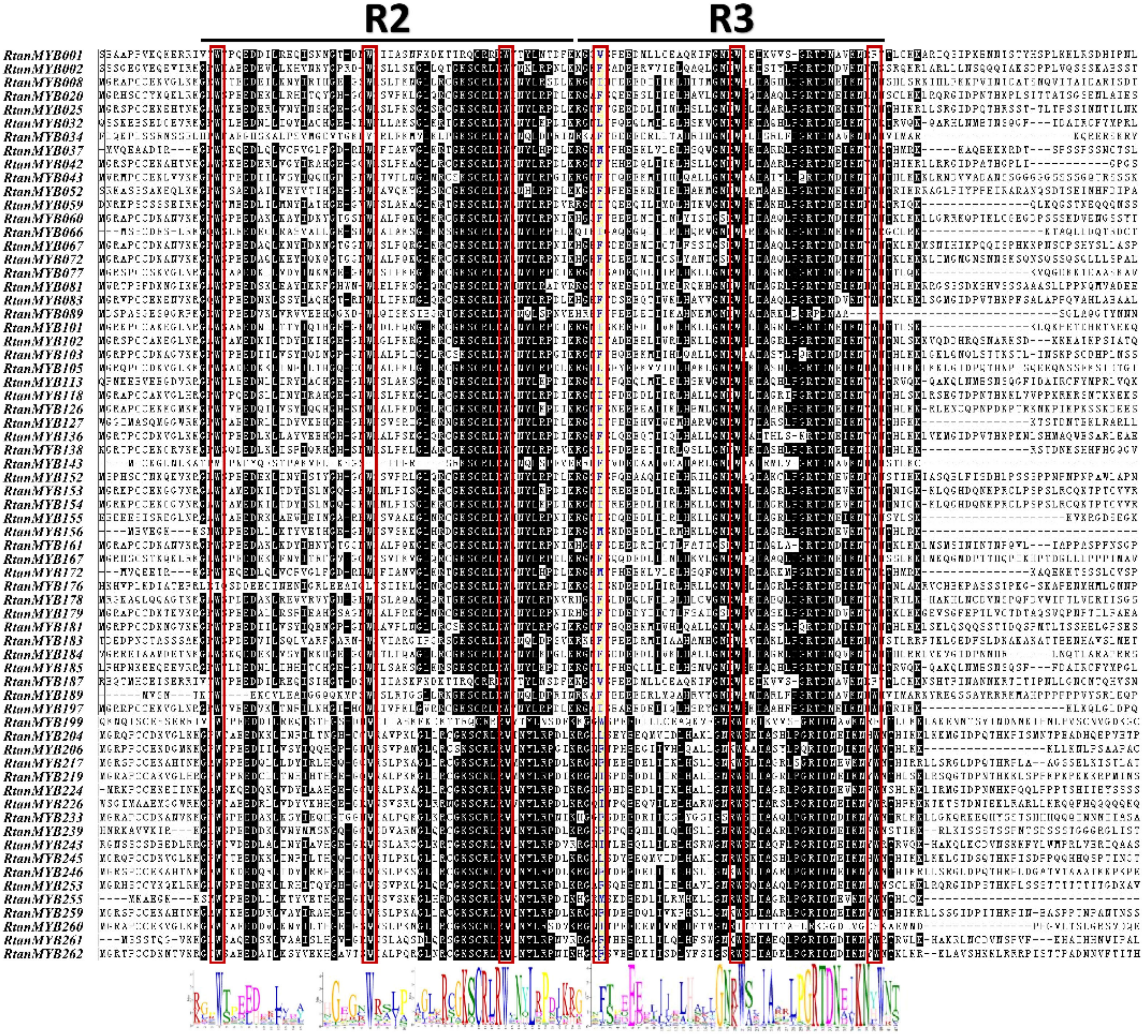
Amino acid sequence analysis of R2R3-MYB TFs in *R. tanguticum* that may be involved in AQs biosynthesis. The conserved sequences of key features are highlighted with a black background, and the conserved “W” in the R2 and R3 domains is marked with a red box.

The phylogenetic relationship of R2R3-MYB TFs in *R. tanguticum* and *Arabidopsis thaliana* was analyzed using MEGA12 (**Figure 2**). These genes were divided into 11 clades (C1-C11) according to the topology of the phylogenetic tree. Based on the TAIR (https://www.arabidopsis.org/) and prior research on *Arabidopsis thaliana* R2R3-MYB TFs, the A1 to A27 subfamilies and potential functions of their genes were indicated in **Figure 2** (Dubos et al., 2010). For instance, the *RtanMYB* TFs in C7 were categorized into groups A19 and A20. Genes in these subfamilies might regulate anthocyanin metabolism and have certain defensive functions. The clustering on the phylogenetic tree of *Arabidopsis thaliana* - based subgroups into larger topological structures might imply convergent evolution of these genes in *R. tanguticum* (C1, C2). Similarly, some genes that originally belonged to the same subgroup lost their original classification trend in topological differentiation, likely due to gene functional divergence (C3 and unshaded branches on the tree).

**Figure 3:**
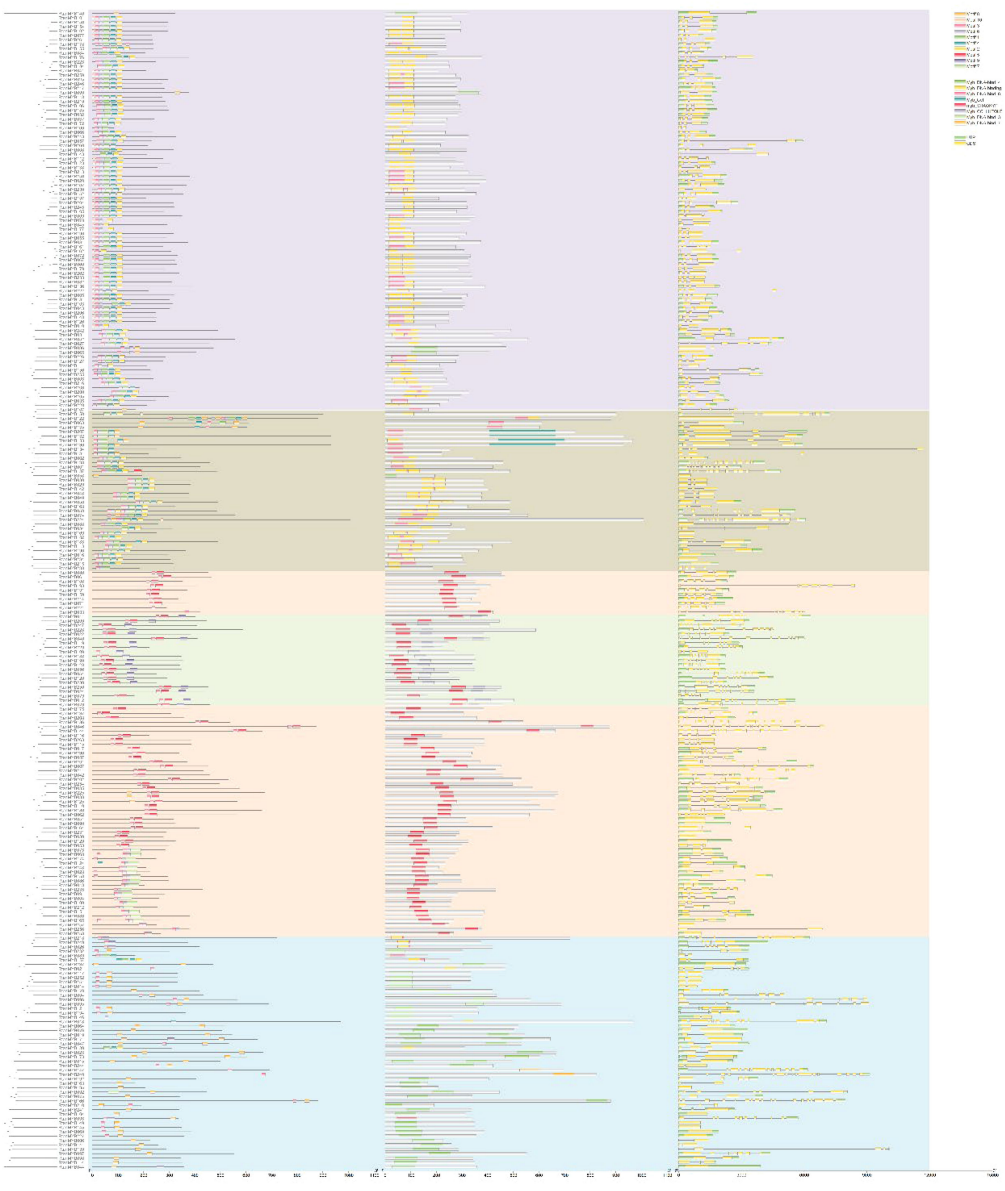
The evolutionary tree, conserved motifs, conserved domains, and gene structure of the 263 MYB TFs in *R. tanguticum*.

CHSs shared between the AQs polyketide pathway and acetate-pathway-derived flavonoids is the breakthrough for mining MYB TFs regulating AQs biosynthesis (Kang et al., 2020; Tang et al., 2024). We screened some subgroups (A1, A3, A6, A7, A9, A10, A14, A17, A18, A19, A20, A27) involved in the regulation of secondary metabolite synthesis (especially the flavonoid pathway) and properly expanded them based on their topological cluster categories (C1, C2, C3, C5, C6, C7, C11), identifying 67 MYB TFs with potential functions. Analysis of the sequences of 67 MYBs shown they generally have typical R2 and R3 domains (**Figure 3**). Moreover, some sequences contain specific conserved motif signatures (bHLH, ANDV/ENVI et al.) for flavonoid pathway metabolites (**Table 2**). Previous studies had demonstrated that certain MYB TFs, which modulated flavonoid biosynthesis, could either activate or inhibit CHSs. These genes not only possessed the typical R2-R3 domain but also contained signature conserved domains such as ENVI, TLLLFR, or SG7. Further screening and analysis based on these conserved domains would lay an important groundwork for identifying MYB TFs that regulated CHSs in the AQs biosynthesis (polyketide pathway).

**Table 2:** Characteristics of motifs in MYB TFs related to AQs based on CHSs (Karppinen et al., 2021).

Motifs	Sequence features	Function/presence
bHLH interaction	[D/E]Lx2[R/K]x3Lx6Lx3R	all MYBs (Except flavonol-regulating MYBs)
ANDV \NDEI	[A/S]NDV\NDE[I/V]	C2 repressors, PA-type MYBs
C1 \C2 \C3 \C4	[K/R]Px3[K/T][F/Y]	C2 repressors, MYB5, PA1-type
TLLLFR	Lx3GIDPx[T/N]H[R/K], pdLNL[D/E]L, dFLGL	subclade D2 of C2 repressors
TT2-box	TLxLF[R/G]	TT2/PA2-type MYBs
PA1	V[V/I]R[T/P][K/R]Ax[R/K]C	PA1-type MYBs
G-28	K[I/V]x2PKPxRx2S[I/L]	PA1-type MYBs
SG7	EKLYEEYLQLLx7-10QLDSFAESLLI	Flavonol-regulating MYBs
SG7-2	[K/R][R/x][R/K]xGRT[S/x][R/G]xx[M/x]K	Flavonol-regulating MYBs

Subsequently, we performed a phylogenetic analysis of MYB TFs across all four species based on the subfamily classification of MYB TFs (**Supplementary Figures S3–S6**). The same method was used to divide different clusters on the phylogenetic tree. Using Arabidopsis thaliana as an internal reference, the contracting and expansion of gene numbers in different subgroups revealed that MYB TFs have undergone different evolutions after the differentiation of species in the *Rheum* genus. Notably, the number of MYB TFs in stress - related subgroups is similar in *R. tanguticum* and *R. nobile*, which may be related to their similar habitats (Chen et al., 2024).

### 3.4 Motif, domain and structure analysis of MYBs in four *Rheum* L. species

More systematic evolutionary relationships among four *Rheum* L. species are revealed through comprehensive sequence analysis. To understand the conserved structural domains of MYB proteins in the four *Rheum.* L species, we conducted Motif Elicitation (MEME) analysis on each identified MYB TFs. Ten different conserved motifs were discovered in the MYB TFs (**Supplementary Figures S7–S10**). Among these motifs, we detected many conserved amino acids, particularly tryptophan residue (W), which was a notable feature of the MYB domain (Wu et al., 2022). It could be observed that closely related genes have similar types and numbers of motifs, but each gene has a different domain structure (**Figure 3**; **Supplementary Figures S11–S13**). Different motifs contained different types of conserved amino acids. This was of great significance for differentiation of protein functions.

*RtanMYB* TFs containing motif 1, motif 4 and motif 7 were closely related (**Figure 3**), indicating that they have evolved distinct characteristic sequences from other MYB proteins during the process of evolution. Similar distribution of motifs among MYB proteins within the same subgroup suggested that motif distribution might be related to function. A similar situation also occured with motif 6 and motif 7 (**Figure 3**). *RobMYB* TFs containing motif 1 and motif 3, *RhpMYB* TFs containing motif 3 and motif 5, and *RnoMYB* TFs containing motif 1 and motif 2 were also the same (**Supplementary Figures S11–S13**). In addition, we observed that motif 3 was present in almost all members of *RtanMYB* TFs, indicating that motif 3 played an essential role in influencing the function of MYB TFs. In *RobMYB* TFs, motif 2 also played the same role, and the same situation occurs in the *R. palmatum* and *R. nobile* (**Supplementary Figures S11–S13**). These motifs might exist as the most conserved core MYB domain-forming motifs.

To elucidate the gene structure of the MYB family, we analyzed the introns and exons of the MYB genes. Within *RtanMYB* TFs (**Figure 3**), MYB genes clustered in the same subgroup have roughly similar exon/intron structures, numbers of exons, and gene lengths. In contrast, other subgroups exhibited greater variation in the number of exons and introns, and this structural diversity of this genes might reflect the evolutionary divergence between homologous MYBs. Furthermore, this could indicate a tendency for genes within a subfamily to evolve conserved functions, while members between subfamilies might undergo more functional differentiation during the evolutionary process. CDS structures of other three *Rheum* L. species also shown similar patterns (**Supplementary Figures S11–S13**).

### 3.5 Analysis of *cis*-acting elements in the promoter region and protein function of MYB TFs

AQs provide photoprotection and enhance disease resistance in plants, which is crucial for high altitude plants. Many studies have reported that environmental conditions affect the activation of MYB TFs regulating plant secondary metabolite synthesis (Ji et al., 2022). Analysis of promoter *cis*-acting elements could provide insights into stress response patterns of genes. 2kb upstream promoter region of MYB TFs was analyzed for CREs. 47 CREs related to plant growth and development and stress response were detected (**Supplementary Table S4**).

From a variety of CREs, five highly significant ones were screened using keywords related to environmental factors and stresses, such as “motifs” and “boxes”. Most of these response elements are MeJA (CGTCA - motif), ABRE, MBS, and LTR. Notably, MeJA has been reported to significantly induce the expression of genes related to AQs biosynthesis and increase AQs content in plants (Tang et al., 2024). Therefore, screening for potential MYB TFs involved in AQs biosynthesis regulation based on the number of CGTCA-motifs in specific MYB TFs is a reliable approach. In this regard, *RtanMYB042*, *RtanMYB131*, *RnoGMYB061*, *RhpMYB063* et al. show great potential.

### 3.6 Chromosomal location, duplication events of MYB genes

Chromosome localization shown that these genes were distributed across 11-12 chromosomes in each species, number and density of MYB genes on each chromosome were unevenly distributed. For example, in *R. tanguticum*, there was a relatively low number of genes found on chromosome 10 (15), while a higher number of genes are found on chromosomes 1 and 3 (>30). Additionally, MYB genes were primarily distributed at the ends of the chromosomes, with fewer in the centromeric regions.

**Figure 4:**
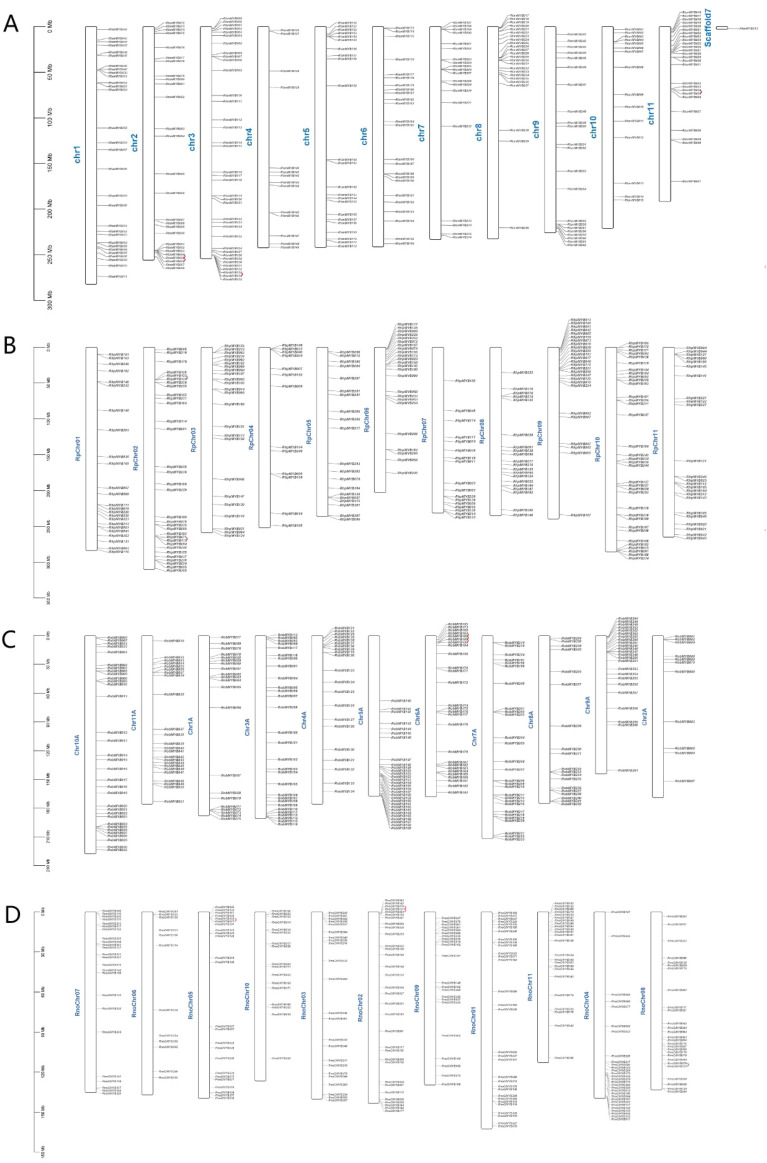
The tandem duplication events and distribution of MYB TFs on the 11 chromosomes of the Rheum genus plants. The scale represents the length of the Rheum chromosomes. **(A)** The tandem duplication events and distribution of *RtanMYB* TFs on the chromosomes; **(B)** The tandem duplication events and distribution of *RhpMYB* TFs on the chromosomes; **(C)** The tandem duplication events and distribution of *RobMYB* TFs on the chromosomes; **(D)** The tandem duplication events and distribution of *RnoMYB* TFs on the chromosomes.

**Figure 5:**
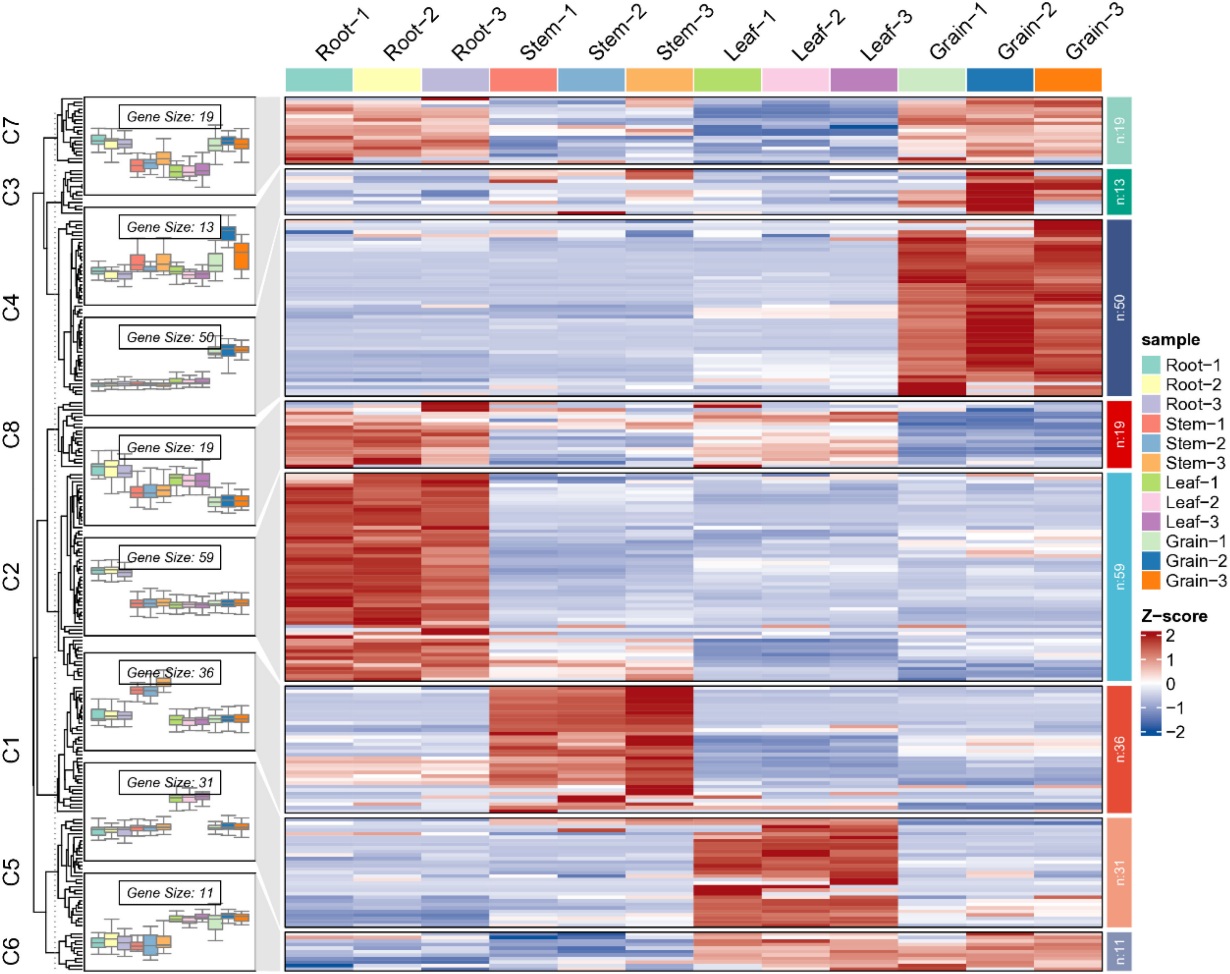
Expression heatmap of candidate genes in different tissues of *R. tanguticum* (A clustering heatmap is constructed using Log2 FPKM values, with further annotation in **Supplementary Table S7**).

Gene duplication contributed to gene amplification during the process of molecular evolution. They could occur through the following mechanisms: segmental duplication, tandem duplication or retroposition (Qiao et al., 2019). 7 tandemly duplicated genes in *R. tanguticum* were distributed across 3 chromosomes (**Supplementary Table S5**), with the highest number of genes found on chromosome 2 (**Figure 4**). Some of these tandemly duplicated genes had been identified as atypical MYBs, and there was also a transition between R2R3-MYB and 3R-MYB. Additionally, 51 pairs of genes related to segmental duplication events were identified, and these genes are similarly dispersed, with members from every subfamily being found. Similar results were also found in other three species (**Supplementary Table S5**; **Figures 4**, **5**). If a gene lacks introns, is polyadenylated at the 3’ end, has short direct repeat sequences at both ends, and is transcribed via mRNA to a chromosomal location different from that of the donor gene, then the gene is considered a retrogene (Liu A. et al., 2020). However, no MYBs were found met these criteria; therefore, it was hypothesized that the MYB TFs in rhubarbs had a low susceptibility to retroposition. Based on aforementioned results, both tandem and segmental duplications might play a key role in the diversity of MYB genes across the four *Rheum* L. species, with segmental duplication being predominant.

**Figure 6:**
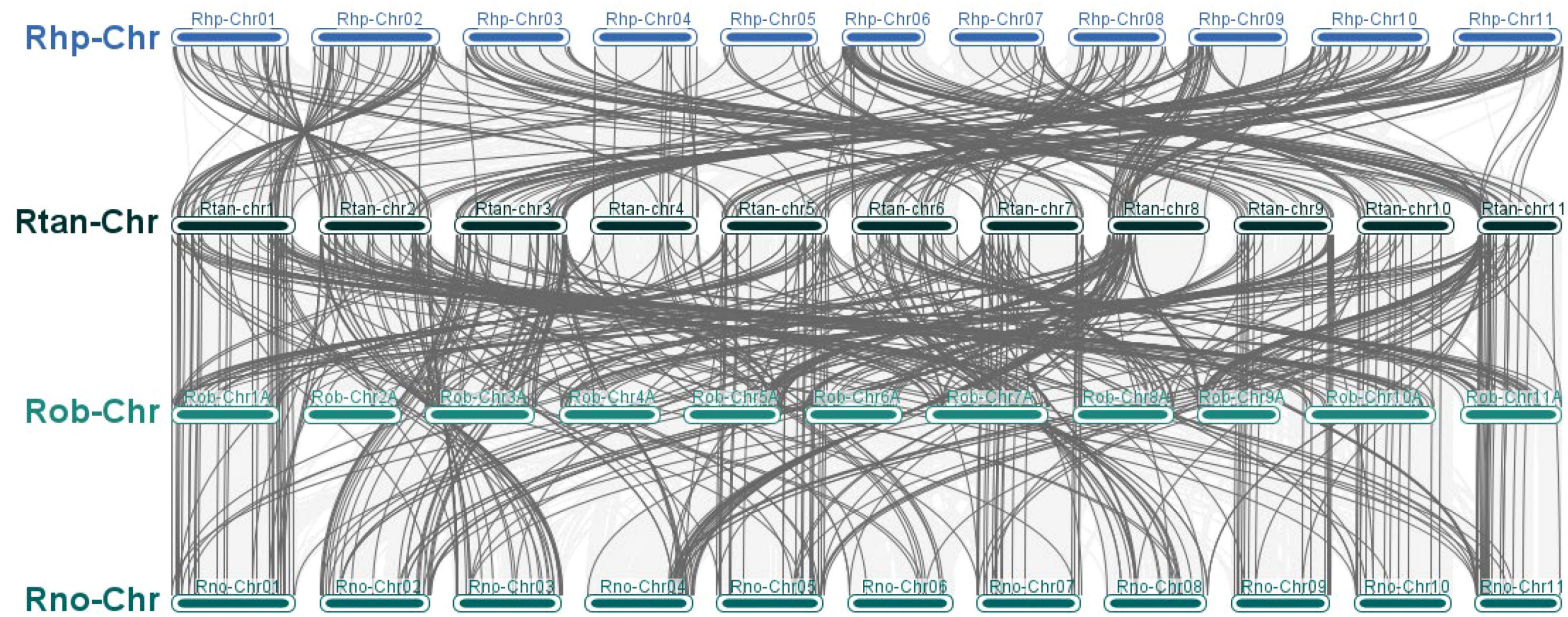
Duplication and synthesis of MYB TFs in *Rheum* L. plants. Comparative physical mapping indicates that *RtanMYB* TFs are homologous to *R. palmatum*, *R. offcinale*, and *R. nobile*.

Some studies hypothesized that the size of a gene family in many species is a infeasible indicator of genetic diversity, which could be applied broadly to many gene families (Simionato et al., 2007). This study illustrated the extent of commonality in MYB gene repertoires between each pair of the four *Rheum* L. species, with the main indicators being the number of MYB orthologs and ATGs in the concerned species (**Figure 6**). For example, there were 223 and 205 orthologs between *R. tanguticum* and *R. palmatum*, with 97 one-to-one orthologs, and between *R. tanguticum* and *R. officinale*, this number was 98 (**Supplementary Table S6**). Therefore, only 36.5% (on average) of MYB genes presented in the most recent common ancestor (MRCA) of *R. tanguticum*, *R. palmatum* and *R. officinale* are conserved in all three species. In contrast, this value is only 33% between *R. tanguticum* and *R. nobile*. However, due to gene duplication events, the ancestral MYB genes in the MRCA have generated 126 and 108 MYB genes in *R. tanguticum* and *R. palmatum* respectively. On average, 73% of the MYB gene repertoires in the four *Rheum* L. plants are shared. Among the six comparisons between the four species, the MYB gene repertoires of *R. tanguticum* and *R. palmatum* are the most similar (86.1%, **Supplementary Table S6**), revealing their closest evolutionary relationship. However, the proportion of shared genes between the other three species pairs is not high (< 80%). Thus, MYB members in the four *Rheum* L. species revealed by this study are genetically diverse.

The ratio of nonsynonymous to synonymous substitutions (Ka/Ks) was an effective indicator for determining positive selection pressure after duplication and was commonly used to understand the direction of evolution of coding sequences and the intensity of selection (Lynch and Conery, 2000). A Ka/Ks ratio of 1 indicated neutral selection, a Ka/Ks ratio less than 1 indicated purifying selection, and a Ka/Ks ratio greater than 1 indicated positive selection (Lynch and Conery, 2000).

To investigate the role of these duplication events in the evolution and functional divergence of *RtanMYB* genes, we calculated Ka, Ks, and Ka/Ks for each gene pair. Among them, the average Ka and Ks values for tandem duplications of *RtanMYB* genes were lower than those for segmental duplications (**Supplementary Table S5**), while the average Ka/Ks of segmental duplication genes was lower than that of tandem duplication, and might be candidate genes that have undergone positive selection (>0.5), but the overall Ka/Ks value of *RtanMYB* genes was less than 0.5 (**Supplementary Table S5**). Similar results were also found in the orthologous gene pairs of *R. palmatum* and *R. offcinale.* The duplication events of segmental and tandem duplication genes in *R. tanguticum* were estimated to have occurred approximately 194 million years ago (Mya) and 47 Mya. These results suggested that these duplication events played an important role in the evolution and functional divergence of *RtanMYB* genes. MYB TFs in *R. palmatum* and *R. offcinale* exhibited the same phenomenon. The divergence times of *R. palmatum* and *R. offcinale* were 210 and 164 Mya, respectively.

### 3.7 Expression profiling of MYB TFs of *R. tanguticum* in different tissues

PCA of gene expression in different parts of *R. tanguticum* shown great repeatability within each group and could clearly distinguish between different parts. On PC1 (Principal Component 1), roots were significantly separated from stems, leaves, and seeds, indicating that genes expressed in the roots have significant differences from those in other parts. On PC2, roots and seeds were similar, and both were significantly separated from stems and leaves (**Supplementary Figure S2B**).

Comparative transcriptome analysis revealed that among these 263 *R. tanguticum* MYB TFs, 21 genes were not detected in expression across the four selected tissues (excluding genes with poor reproducibility). Remaining *RtanMYB* TFs were expressed in at least one tissue, with no genes found to be highly expressed in all four components simultaneously. There were 97 up-regulated and 78 down-regulated differential expression MYB genes (DEMGs) in the root compared to stem and leaf samples; In comparison with the seed samples, there were fewer up-regulated DEGs, totaling 78, and a higher number of down-regulated DEMGs, amounting to 93 (**Figure 5**; **Supplementary Table S7**). Clustering diagram shown different expression levels among these four groups (**Figure 5**). Clustering analysis results divided these genes into 8 groups, whose expression profiles in each component did not have universal similarity, except for MYB TFs in the root and seed, which have a more similar expression profile in C7 and are clustered in a branch. In other clusters, there are significant differences in the expression levels of DEGs, with 42, 97, 82 and 36 highly expressed genes in leaves, roots, seeds, and stems respectively (**Figure 5**; **Supplementary Table S7**).

The expression divergence of duplicated genes typically occured after functional differentiation (Duarte et al., 2006). This study analyzed the expression differences between MYB genes that underwent duplication events. The divergence in their clustering pattern diagram suggested a high degree of variability in their expression patterns, indicating that the expression patterns of most gene pairs tended to be uncorrelated in the subject tissues (**Supplementary Tables S5, S7**; **Supplementary Figure S8**). Additionally, it was found that there was a phenomenon where the expression level of one gene in a pair of genes was higher than that of the other (**Supplementary Tables S5, S7**; **Supplementary Figure S8**). Many studies had demonstrated that tissue-specific expression divergence was one of the most critical indicators of functional differentiation of duplicated genes, and this divergence usually increased expression diversity and led to the evolution of subfunctionalization or neofunctionalization (Li et al., 2005). Although many duplicated genes shown very low expression values, most of them tend to exhibit tissue-specific expression (**Supplementary Tables S5, S7**; **Supplementary Figure S8**). Therefore, the expression of MYB genes produced by tandem duplication and segmental duplication exhibited divergence, which might herald the emergence of new biological functions after gene duplication events, facilitating the regulation of various physiological processes.

### 3.8 Analysis of AQs accumulation pattern and gene co-expression in different tissues of *R. tanguticum*

**Figure 7:**
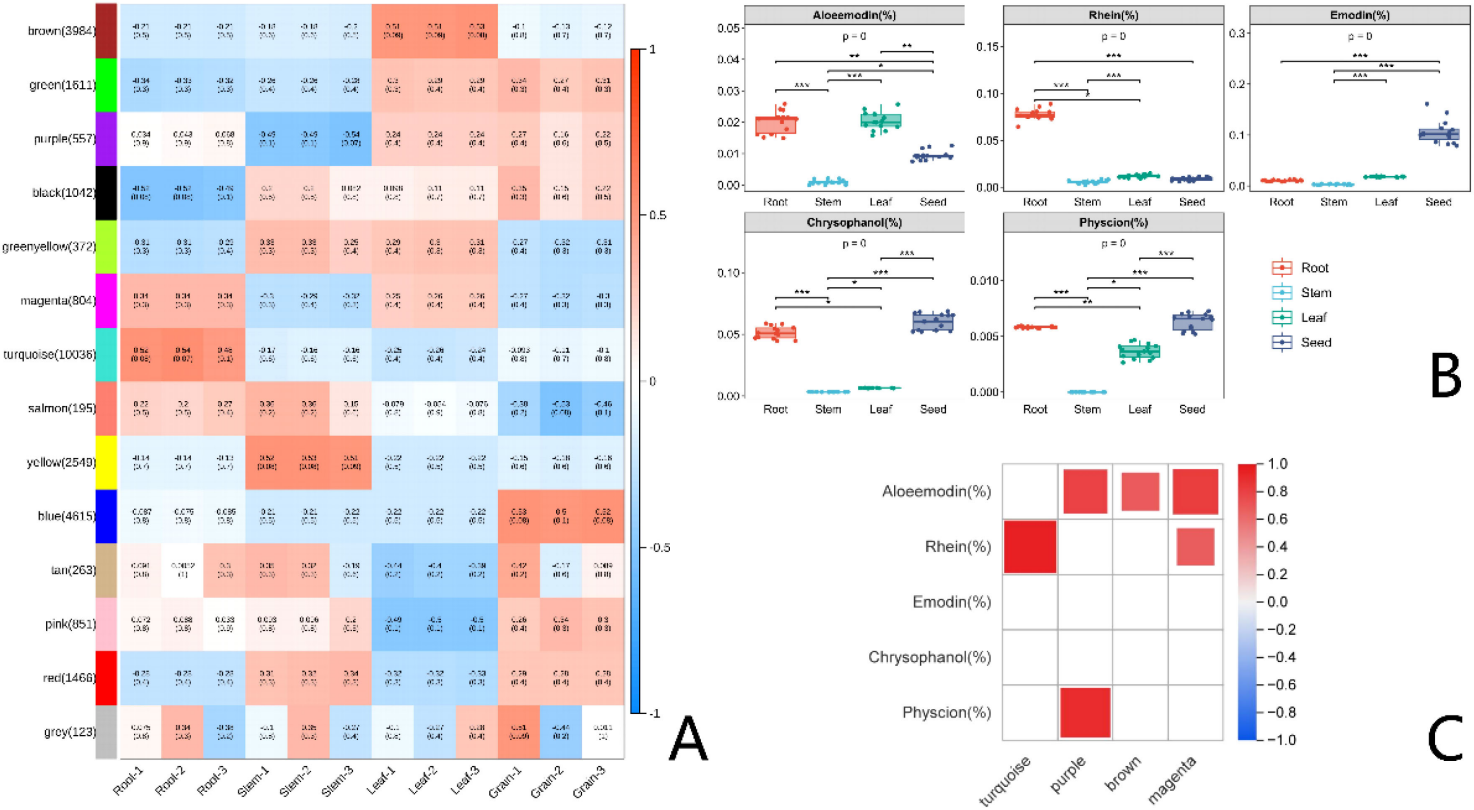
The correlation analysis of *RtanMYB* TFs in *R. tanguticum*. **(A)** Weighted Gene Co-Expression Network Analysis. **(B)** Quantitative analysis (HPLC) of AQs in different components of *R. tanguticum*. **(C)** The Pearson’s correlation coefficients of AQs with MYBs possibly involved in AQs biosynthesis.

**Figure 8:**
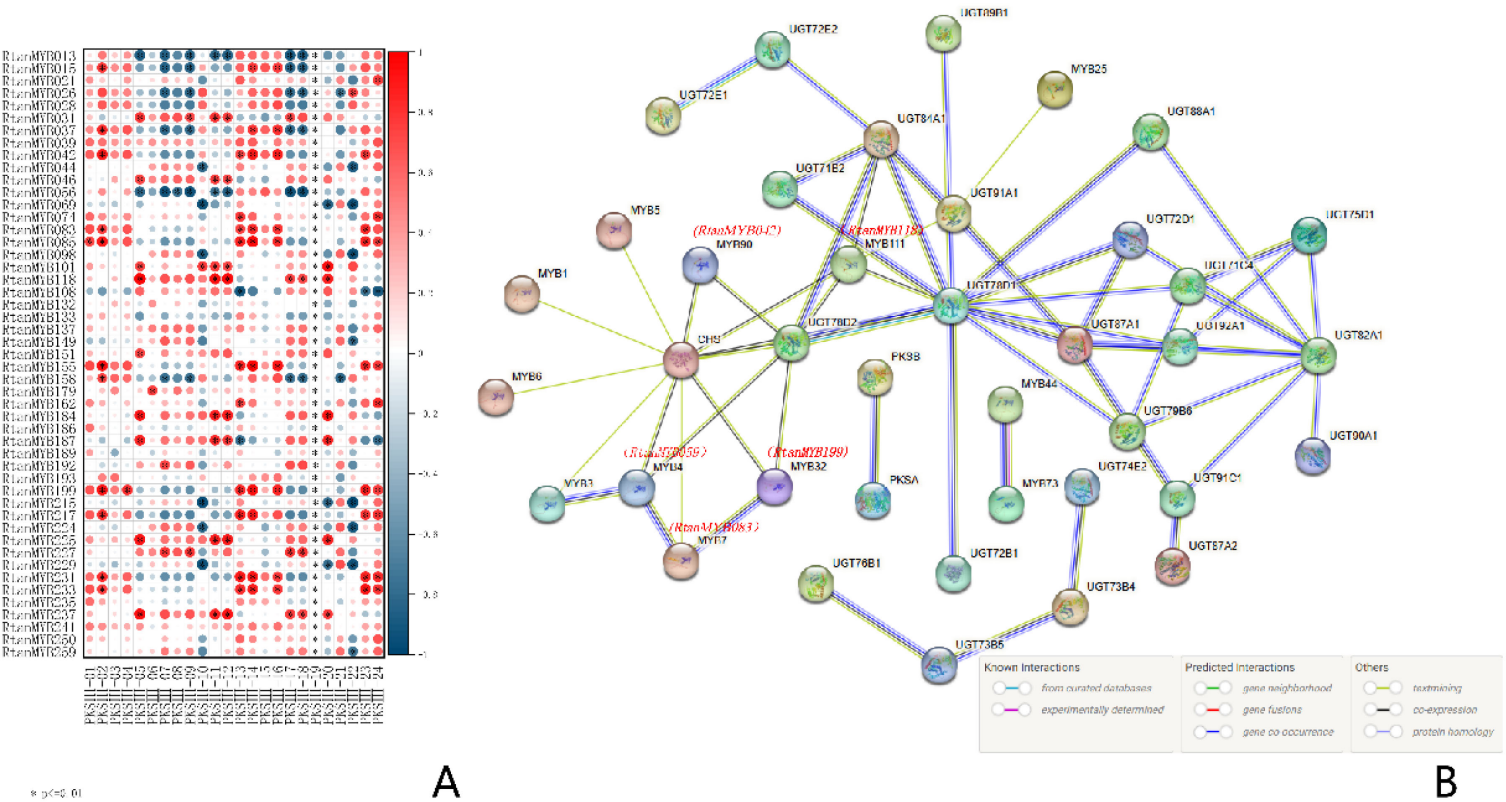
The expression analysis of *RtanMYBs*. **(A)** The characterization of Pearson correlation coefficients between PKS genes involved in AQs biosynthesis and MYB TFs (*p<=0.01). **(B)** The protein-protein interaction simulation of 12 *RtanMYB* TFs with the PKS and UGT gene families.

To investigate the degree of correlation between tissue-specific gene expression and tissue-specific accumulation of AQs, we employed HPLC for quantitative analysis of AQs distribution across various plant components. The results of HPLC analysis showed that, except for Emodin, the other four AQs had higher contents in the roots, followed by leaves and seeds. The content of Emodin was the highest in seeds. Moreover, signals for the five AQs were not detected in the stems (**Figure 7B**). Considering that AQs compounds mainly accumulate in roots, seeds and leaves of rhubarbs, MYB TFs that highly expressing in roots, seeds, and leaves in medicinal plants were most likely involved in regulating the biosynthesis of AQs. WGCNA analysis revealed four typical tissue-specific gene expression modules, with genes in these modules highly expressed in roots, leaves, and seeds (**Figure 7A**). The Pearson correlation characterization of the tissue-specific expression of genes within the four modules and the accumulation patterns of the five AQs further confirmed the strong correlation between these highly expressed genes and AQs accumulation. Among them, 49 MYB TFs (Size > 750 bp) were selected (**Figure 5**; **Supplementary Table S7**). To further investigate and confirm *RtanMYB* TFs related to the AQs biosynthetic pathway, we visualized the correlation between *RtanMYB* TFs and genes encoding key enzymes involved in AQs biosynthesis (**Figure 8A**; **Supplementary Figure S2A**). The results shown that *RtanMYB059*, *RtanMYB083*, *RtanMYB113*, *RtanMYB118*, *RtanMYB183*, *RtanMYB224*, *RtanMYB245*, *RtanMYB042*, *RtanMYB085*, *RtanMYB155*, *RtanMYB199* and *RtanMYB217* exhibited strong potential to be involved in AQs synthesis.

### 3.9 Protein-protein interaction network of MYB TFs involved in the biosynthesis of AQs in *R. tanguticum*

Employing the STRING database, we further predicted the regulatory networks of the candidate MYB TFs and the relevant genes in their protein-protein interaction networks, as illustrated in **Figure 8B**. Studies had shown that CHS and UGT were involved in the biosynthesis of AQs in plants (Mund and Čellárová, 2023). PPI (Protein-Protein Interaction) simulations for these selected genes revealed that CHS and UGT (UDP-Glycosyltransferase), as central hub proteins, had widespread interaction phenomena with the screened *RtanMYB* TFs. This result indicated that there might be multiple pairs of interacting proteins in the AQs biosynthesis regulatory network of *R. tanguticum*. Centered on CHS, 8 MYB TFs were found to interact with it. Notably, *RtanMYB118* not only interacted with CHS but also with 4 UGT genes, suggesting its general role in this regulatory network. *RtanMYB042*, *RtanMYB059*, and *RtanMYB119* also demonstrated similar potential. We speculated that *RtanMYB* TFs might regulate the accumulation of AQs through these two enzyme genes.

### 3.10 Expression verification of candidate MYB TFs in *R. tanguticum*

**Figure 9:**
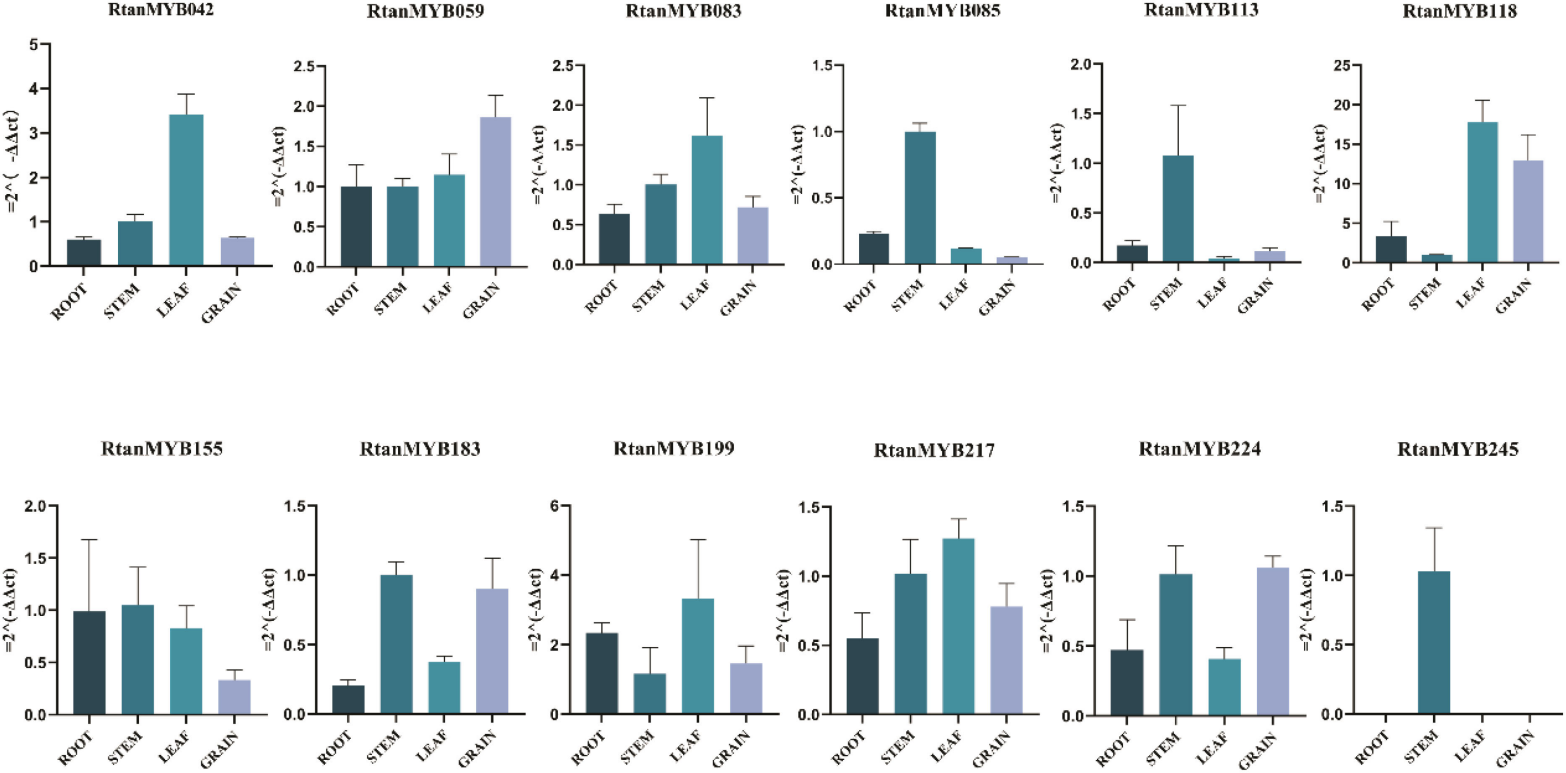
The qRT-PCR of 12 candidate *RtanMYB* TFs (n=3).

Transcription factors regulated growth, development, and secondary metabolism through their own transcriptional activity. Previous work had provided ample evidence for the involvement of these 12 *RtanMYB* TFs in the biosynthesis of AQs, and qRT-PCR experiments further validated the expression of these 12 transcription factors under actual conditions (**Figure 9**). The results shown that *RtanMYB118* and *RtanMYB199* were highly expressed in all components except the stem, while the other 10 *RtanMYB* TFs were highly expressed in one or two components, consistent with the transcriptome data. Previous studies had indicated that AQs mainly accumulate in the roots of *R. tanguticum*, considering this situation, there might be a “biosynthesis-transport” scenario in the accumulation of AQs in *R. tanguticum.* Subcellular localization analysis of *RtanMYB118* and *RtanMYB199* shown that they were nuclear-localized (**Supplementary Table S1**). Therefore, *RtanMYB118/199* was confirmed to play an important role in the biosynthesis of AQs in *R. tanguticum*.

## 4 Discussion

Rhubarbs contains three important *Rheum* L. species that rich in AQs compounds with significant pharmacological activities (Xiang et al., 2020; Zhao S. et al., 2023). In recent years, research on them mainly focused on the extraction of chemical components (Lu et al., 2024). Due to limitation of genomic information, structural genes and transcription factors that play key roles in biosynthesis of AQs are still unclear (Wang et al., 2023). MYB TFs play a crucial role in regulating plant physiological activities (Ma et al., 2023). They had increasingly attracted the attention of researchers in various plant species and has been extensively studied in model plants such as *Arabidopsis thaliana* (Yanhui et al., 2006), *Oryza sativa* (Katiyar et al., 2012), *Solanum lycopersicum* (Guo et al., 2022), as well as in many other species, including *Morella rubra* (Cao et al., 2021). Given the important regulatory role of MYB genes in plant physiology, understanding these genes is crucial for plant research, including breeding and crop improvement. Given the critical regulatory roles of MYBs in plant physiology, understanding these genes is essential for plant research, including breeding and crop improvement. At present, research on whole-genome MYB TFs of rhubarbs and their involvement in AQs biosynthesis is still quite limited. However, studies on the evolutionary relationships and functions of transcription factors using multi-omics approaches in other species have become relatively mature (Hao et al., 2023; Tao et al., 2018). This provides valuable experience for further research into the molecular mechanisms of AQ biosynthesis mediated by MYB TFs in rhubarbs.

### 4.1 Analysis of evolution and expansion of MYB TFs

Studies had shown that plant genomes typically evolve more rapidly than those of other eukaryotes, leading to greater genomic diversity even among closely related plant species (Kejnovsky et al., 2009). In this study, analysis of 1054 MYB genes identified from genomic sequences, as well as 197 MYB TFs from *Arabidopsis thaliana*, revealed that the difference in the number of MYB genes among the four *Rheum* L. species was not very large, and the number of genes did not have a linear correlation with genome size, but it did support the notion of genomic diversity mentioned above (**Supplementary Table S2**). Phylogenetic analysis can provide a clear picture of the evolutionary history of MYB TFs (Li et al., 2021). Evolutionary analysis indicated that MYB TFs from four species can be divided into four groups, with 1R-MYB and R2R3-MYB groups further divided into different subclasses (**Supplementary Figures S3**, **S4**). Within the (sub)groups, there was a significant variation in gene numbers between different species, a phenomenon particularly pronounced in the R2R3-MYB group. The differences in group division and the number of genes within each group reflect the evolutionary patterns of different species. Additionally, a decrease in gene pairs between *R. tanguticum* and *R. palmatum*, *R. tanguticum* and *R.nobile*, was observed. Considering the geographical distribution and cultivation status of these three species, this might be due to differences in domestication. Such differences could further shape the functional divergence of MYB TFs within different groups.

Introns offer insights into the evolutionary trajectories of genes and their corresponding proteins (Li et al., 2024). Their relative positioning can further drive the diversification of gene families (Li et al., 2024). MYB TFs in *R. tanguticum* exhibit variation in intron numbers, suggesting that MYB genes have undergone intron gain or loss during evolution, potentially leading to functional divergence (**Figure 3**). Research indicated that genes with a lower intron count may correlate with higher expression levels (He et al., 2021). qPCR validation of *RtanMYB* genes, which were identified as having higher expression levels in the transcriptome profile, supports this hypothesis (**Supplementary Table S7**; **Figure 9**).

### 4.2 Gene duplication events play important roles in MYBs family expansion

**Figure 10:**
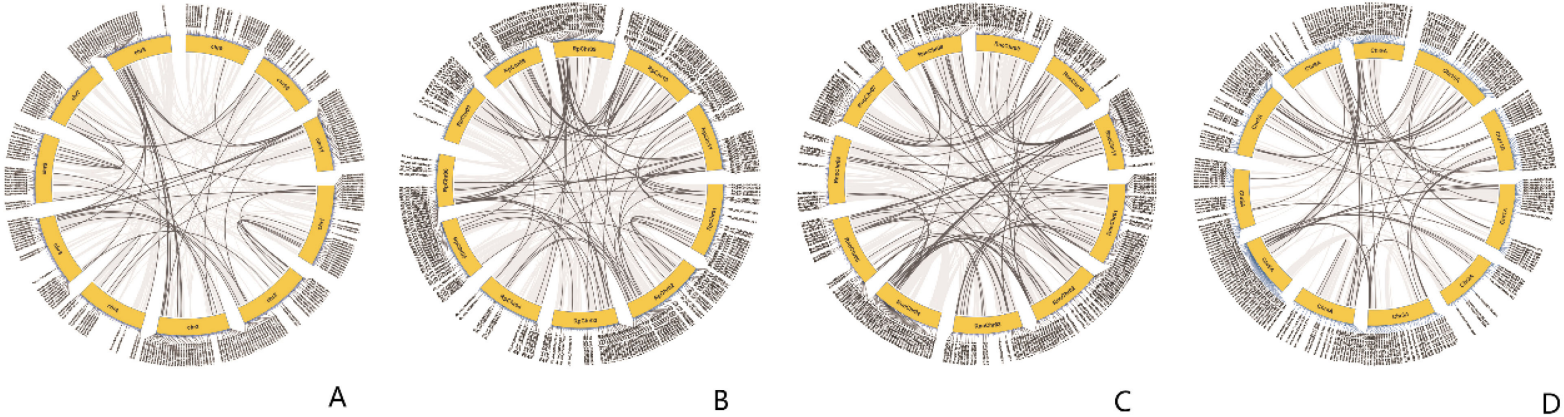
Analysis of duplication events in MYB TFs of *Rheum* L. plants. **(A)** Segmental duplication events of MYB TFs in *R. tanguticum*; **(B)** Segmental duplication events of MYB TFs in *R. palmatum*; **(C)** Segmental duplication events of MYB TFs in *R. nobile*; **(D)** Segmental duplication events of MYB TFs in *R. offcinale*.

Gene duplication events are considered an important mechanism leading to the expansion of gene families and are prevalent in plant genomes (Panchy et al., 2016). Synteny analysis helps to identify the functional and evolutionary relationships of genes within the same species or between different species. This study reveals that multiple paralogous gene clusters (52 pairs) and tandem duplicated gene clusters (4 pairs) in *R. tanguticum* originated from segmental and tandem duplications (**Figures 4**, **10**). Among them, segmental duplication plays a larger role in the expansion of MYB TFs, a phenomenon widely recognized in the 1R-MYB and R2R3-MYB subfamilies. Similar situations also exist in *R. palmatum*, *R. officinale*, and *R. nobile*. Since duplicated genes always tend to undergo subfunctionalization or neofunctionalization to reduce functional redundancy, there may be expression differences between duplicated genes (Duarte et al., 2006). Therefore, new and old duplicated genes often have significant differences in epigenetic modifications, expression patterns, and other aspects, which are related to the functional divergence in expression. For transcription factors, this also leads to the rewiring of transcriptional regulatory networks and changes in transcription factor complexes. Therefore, studying the protein interactions of MYB TFs that have undergone duplication events is of great significance for exploring their functions. Most of the duplicated *RtanMYB* genes from tandem or segmental duplications tend to be unrelated in the four tested tissues, and the expression value of one gene in the tissues is much greater than that of the other gene in the pair (**Supplementary Tables S5, S7**; **Supplementary Figure S8**; **Supplementary Table S7**). This expression divergence can, to some extent, increase the diversity of expression patterns and promote the subfunctionalization or neofunctionalization of duplicated genes.

By constructing collinearity maps among the four *Rheum* L. species, the evolutionary relationships of MYB genes within these species were analyzed. The collinearity maps shown that the number of gene pairs between *R. tanguticum* and the other three species (*R. palmatum*, *R. officinale*, and *R. nobile*) are 103, 91, and 101, respectively. The variation in the number of collinear gene pairs, including the phenomenon of certain gene pairs specifically appearing between certain *Rheum* L. species (for example, *RtanMYB079*/*098*), reflects the conservation status between the two species and indicates that new duplication events have occurred within the MYB gene family during the divergence of these species. Notably, some MYB TFs were found to be associated with at least two collinear gene pairs, suggesting that these genes may be crucial for the evolution of the MYB gene family (**Supplementary Table S6**).

Ka/Ks analysis indicates that within or between four species of this four *Rheum* L. genus, MYB TFs have mostly undergone purifying selection inside the duplicated genomic elements during the process of speciation. Few of these genes have been subject to positive selection (**Supplementary Table S5**). These results suggest that MYB genes in four *Rheum* L. genus have experienced strong purifying selection during their evolutionary process, undergoing minor changes after duplication. In previous studies of the MYB gene family, such as *G. hirsutum* (Salih et al., 2016) and *H. coronarium* (Abbas et al., 2021), a high proportion of purifying selection and a low proportion of positive selection have also been reported.

### 4.3 Some *RtanMYB* TFs play critical roles in the synthesis of AQs

Structural genes encoding compound synthesis enzymes play an important role in the biosynthesis of plant secondary metabolites (Golovko, 2023). Although AQs biosynthesis has been studied in some medicinal plants, their biosynthetic pathways and regulatory mechanisms are not yet fully elucidated. Our previous studies comprehensively confirms that DAHPS, DHQS, DHQD/SDH, SMK, EPSP, CS, ICS, PHYLLO, MenE, and MenB are involved in the shikimate pathway, producing the precursor 1,4-dihydroxy-2-naphthoyl-CoA, and in the polyketide pathway, PKSIII and PKC are involved in the synthesis of AQs precursors. In *R. tanguticum*, PKSIII and UGT genes have been confirmed to participate in AQs biosynthesis. We have screened these enzyme genes from the whole-genome data of *R. tanguticum*, including those with completed functional validation and pending validation, and analyzed their transcriptome profiles (**Supplementary Figure S2A**).

CREs are crucial for controlling transcriptional regulation of various biological processes, one of which is the synthesis of plant secondary metabolites (Li et al., 2024). Existing research has reported that the promoter regions of 24 PKSIII genes and 97 UGT genes involved in AQs biosynthesis in *R. tanguticum* universally contain MYB binding sites (**Supplementary Figure S1**). Correlation analysis based on the Pearson index indirectly confirms this conclusion, indicating that genes for enzymes involved in the upstream and downstream of AQs biosynthesis generally exhibit strong positive or negative correlations with *RtanMYB* TFs. (**Supplementary Figure S2A**).

There have been no reports on the involvement of MYB TFs from closely related species of *R. tanguticum* in AQs biosynthesis. Therefore, this study successively carried out phylogenetic analysis, sequence analysis, and gene family evolution analysis. Based on the quantitative study of tissue-specific accumulation of AQs, tissue-specifically expressed *RtanMYB* TFs were screened. These were then correlated with the 24 PKSIII genes that have been confirmed to participate in AQs biosynthesis (**Figure 8B**). The results shown a strong response from 12 *RtanMYB* TFs, including *RtanMYB118* and *RtanMYB199*.

The protein interaction results of the 12 *RtanMYB* TFs simulated in *Arabidopsis thaliana* shown that homologs of these *RtanMYB* TFs interacted with the central UGT and CHS proteins. CHS and UGT are both key enzyme genes involved in plant AQs biosynthesis, and we speculate that they may regulate the accumulation of AQs through CHS and UGT.

The RNA-seq and qRT-PCR results are consistent at plant tissue level. Based on the RNA-seq results, the expression levels of some genes are relatively high in roots, leaves, and seeds, especially *RtanMYB118* and *RtanMYB199*. RT-qPCR shows that *RtanMYB118* and *RtanMYB199* exhibit the same expression pattern (**Figure 9**). Subcellular localization results indicate that both *RtanMYB118* and *RtanMYB199* have nuclear localization signals, which further confirms their transcriptional regulatory functions in AQs biosynthesis. Tissue-specific expression may confer different biological functions on family members. Functionally similar genes are closely related in the evolutionary process and often have high homology; the similarity in the expression patterns of *RtanMYB118* and *RtanMYB199* can explain the consistency of their functions.

Based on the evidence presented, *RtanMYB118/119* can serve as important targets for research on AQs biosynthesis and subsequent molecular breeding of *R. tanguticum*.

## 5 Conclusions

In summary, through comparative genomic analysis, 1054 MYB TFs were identified and characterized. Comparative analysis of molecular features and extensive phylogenetic analysis revealed the genetic diversity of MYB TFs in four *Rheum* L. plants. Traditional classification and optimization of the MYB TFs were completed based on sequence and evolutionary features, with four *Rheum* L. species of different medicinal value and domestication levels showing significant expansion or loss to varying degrees. Tandem and segmental duplications both contributed to the expansion of the MYBs, but segmental duplication events were markedly dominant. Expression profiles of four tissues revealed that duplicated genes generally exhibit divergent expression patterns, implying a tendency towards neofunctionalization or subfunctionalization after duplication. A detailed analysis of the expression of MYBs in different tissues allows us to identify DEGs in these tissues, and this data will make a significant contribution to future functional genomics research. In addition, this study also discovered and confirmed *RtanMYB118/199*, which affects the biosynthesis of AQs. In summary, phylogenetic analysis has laid the foundation for further research on the physiological functions of MYB TFs in rhubarbs; the MYB TFs in rhubarbs play an important regulatory role in the biosynthesis of AQs, providing an important research target for the molecular breeding studies of rhubarbs as a medicinal resource.

## Data Availability

Publicly available datasets were analyzed in this study This data can be found here: China National Center for Bioinformation (CNCB, https://www.cncb.ac.cn/) repository, with accession number PRJCA037598, PRJCA012373 and CNP0003451. National Center for Biotechnology Information (NCBI, https://www.ncbi.nlm.nih.gov/), with accession number PRJNA719574, PRJNA735904, PRJNA827652, and PRJNA1049137. The assembled genome and the genome annotation are available at figshare database (https://doi.org/10.6084/m9.figshare.25495309).

## Supplementary material

**Additional file 1**: **Supplementary Figure S1**. The PCA analysis of unigenes based on the expression levels in four major components of R. tanguticum; each treatment was performed in three replicates. **Supplementary Figure S2**. The characterization of Pearson correlation coefficients between genes related to the shikimate pathway in AQs biosynthesis and MYB TFs (*p<=0.01). **Supplementary Figure S3-S6**. The phylogenetic tree of MYB TFs subfamily classification system for four Rheum species and Arabidopsis (1R-MYB, R2R3-MYB, 3R-MYB, 4R-MYB). **Supplementary Figure S7-S10**. The protein conserved motifs of MYB TFs in Rheum plants. **Supplementary Figure S11-S13**. Phylogenetic analysis, motif composition, conserved domains, and gene structure of MYB TFs in Rheum plants.

**Additional file 2:** **Supplementary Table**. The basic data on the screened MYB TFs from Rheum plants and their classification and analysis.
