# Supplementary material for: Genome-wide analysis of MYB transcription factors in four Rheum L. plants provides new insights into the synthesis of Anthraquinones
Source: Front Plant Sci. 2025 May 16;16:1558321. doi: 10.3389/fpls.2025.1558321 (PMC12122507; doi:10.3389/fpls.2025.1558321)
Supplement: Supplementary file 2 [file DataSheet1.pdf]

Genome-wide analysis of MYB transcription factor families in four *Rheum* L. plants provides new insights into the synthesis of anthraquinones

Tao WANG[1,2+], Shuo ZHAO[1,2+], Bo WANG[1,2], Jianan LI[1,2], Zengrong YE[1,3], Famei ZHANG[1,2], Huiyuan MA[1,4], and Guoying Zhou[1,2*]

[1] Key Laboratory of Tibetan Medicine Research, Northwest Institute of Plateau Biology, Chinese Academy of Sciences, Xining 810008, Qinghai, People's Republic of China

[2] University of Chinese Academy of Sciences, Beijing 100049, Beijing, People's Republic of China

[3]College of Agriculture and Animal Husbandry, Qinghai University, Xining 810016, China

[4]College of Life Sciences, Qinghai Normal University, Xining 810016, China

[+]These authors have contributed equally to this work

*Corresponding author: Dr. Guoying Zhou, Tel: +86-971-6159630, Fax: +86-971-6143282, E-mail: zhougy@nwipb.cas.cn

ORCID: https://orcid.org/0000-0003-2485-6172

Address: 23# Xinning Road, Xining, Qinghai, P. R. China 810008

Abbreviation *Rheum tanguticum* Maxim.ex Balf: *R. tanguticum*, *Rheum palmatum*: *R. palmatum, Rheum offcinale* Baill: *R. offcinale, Rheum nobile* Hook. f. & Thomson: *R. nobile*

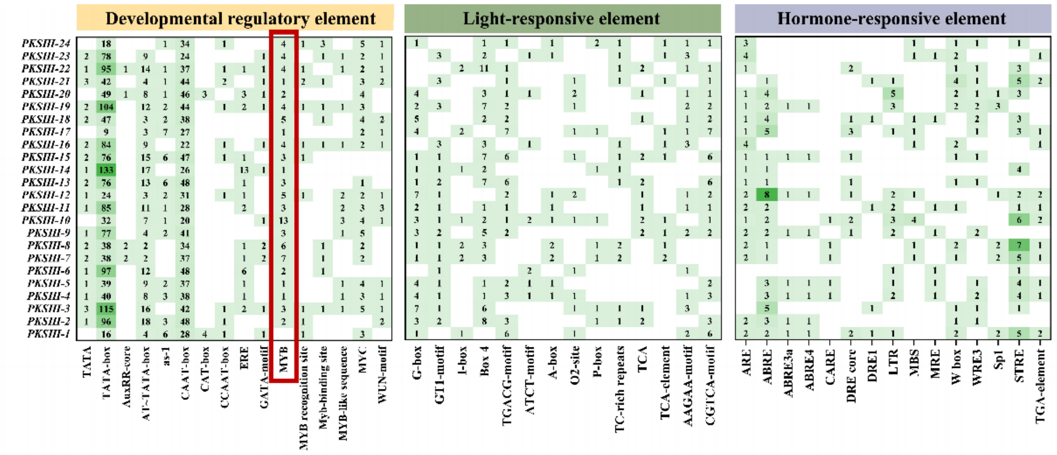

Fig. S1. Analysis of cis-acting elements of the 24 PKSIII genes. The binding sites of MYB TFs are marked with red boxes (Zhao S., 2024b).

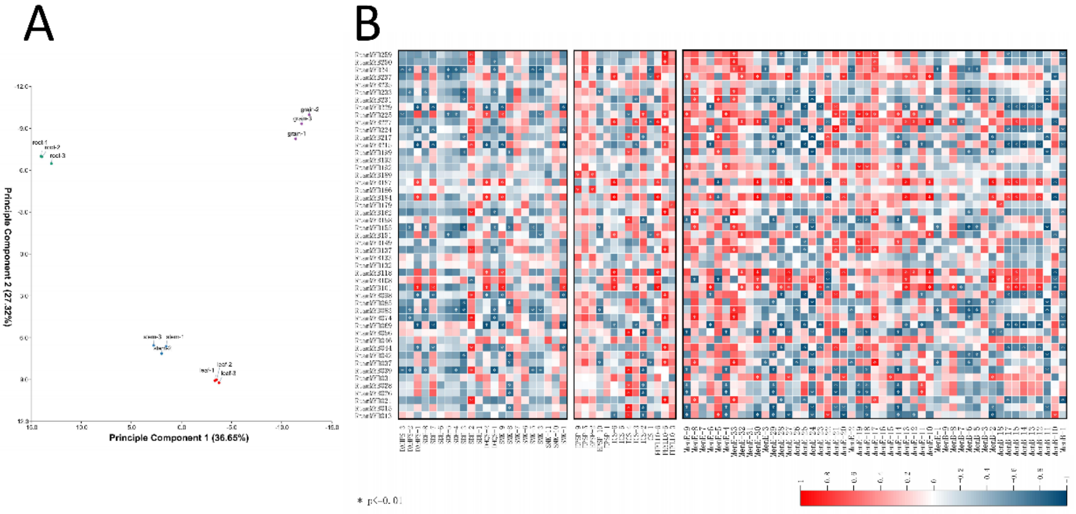

Fig. S2. (A) The PCA analysis of unigenes based on the expression levels in four major components of R. tanguticum; each treatment was performed in three replicates. **(B)**The characterization of Pearson correlation coefficients between genes related to the shikimate pathway in anthraquinone biosynthesis and MYB TFs (*p<=0.01).

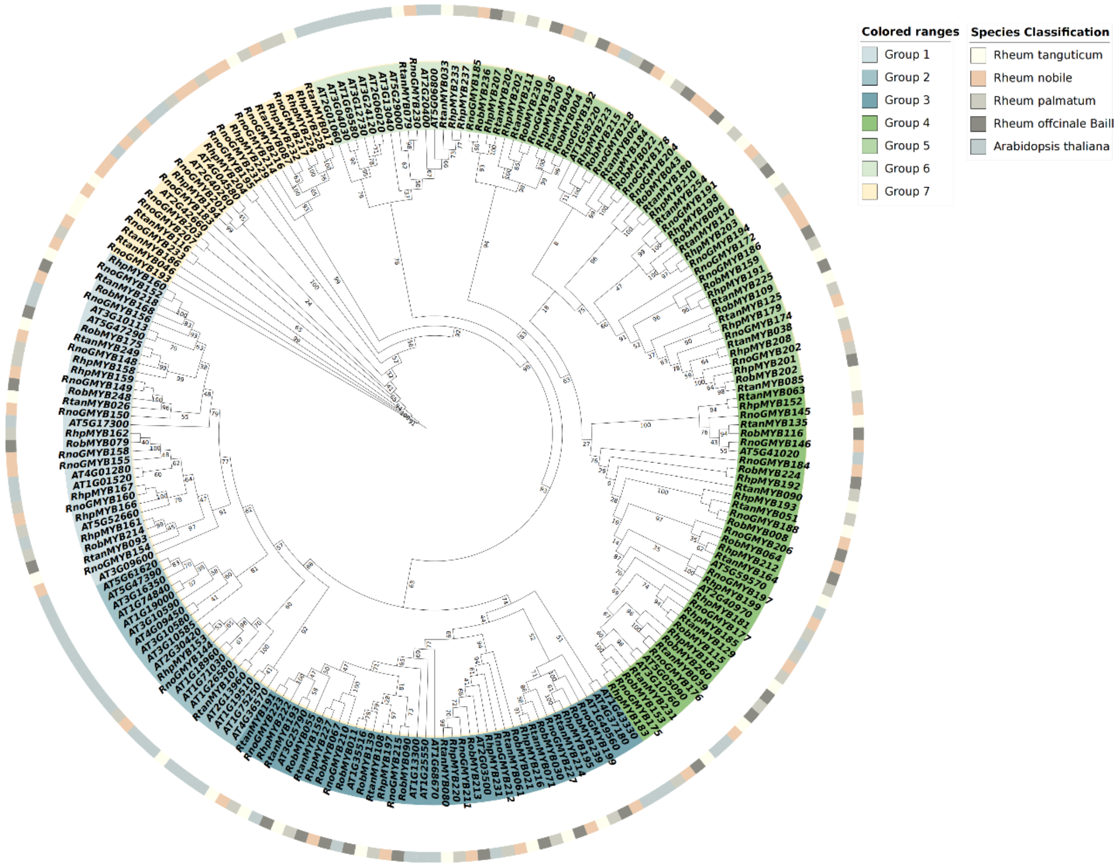

Fig. S3. The phylogenetic tree of MYB TFs subfamily classification system for four Rheum species and Arabidopsis (1R-MYB).

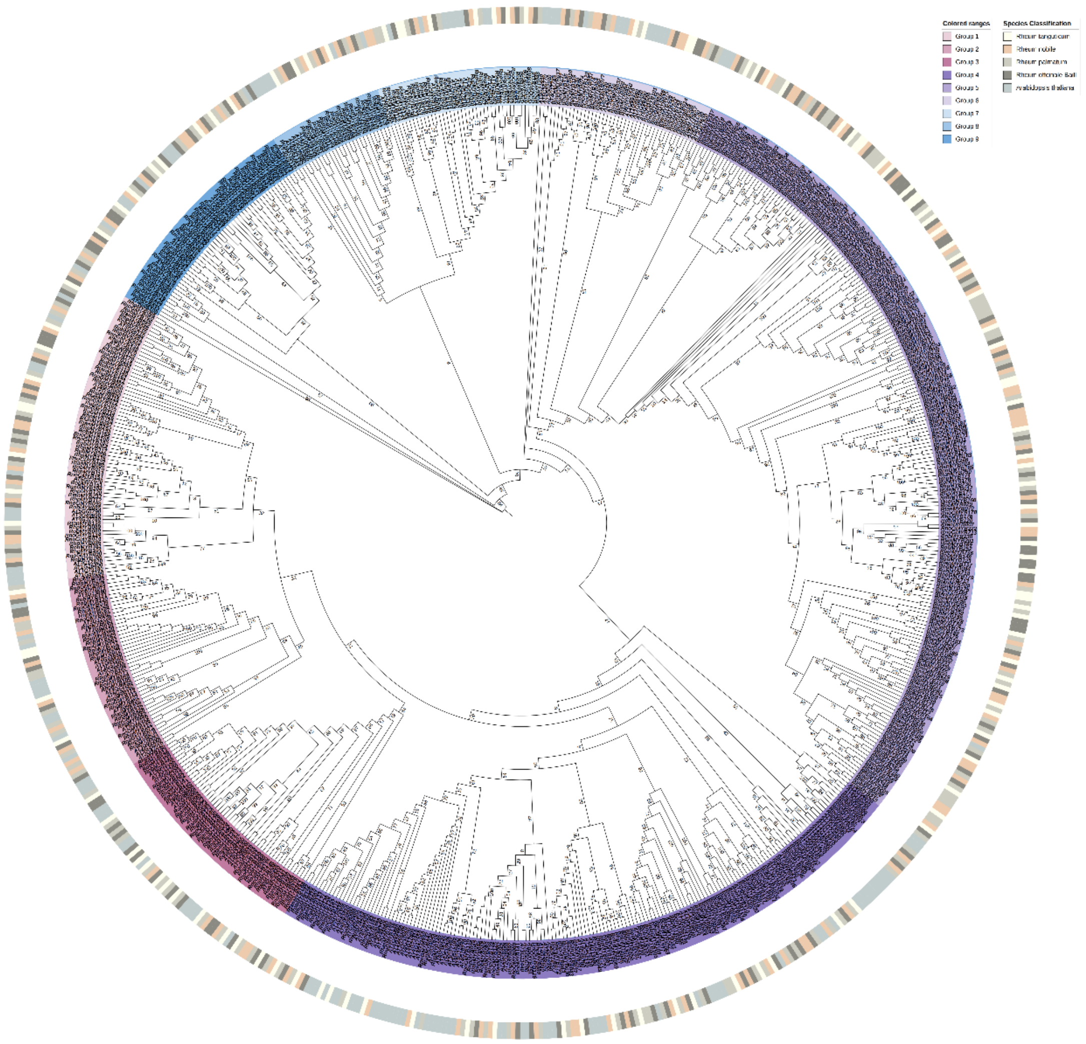

Fig. S4. The phylogenetic tree of MYB TFs subfamily classification system for four Rheum species and Arabidopsis (R2R3-MYB).

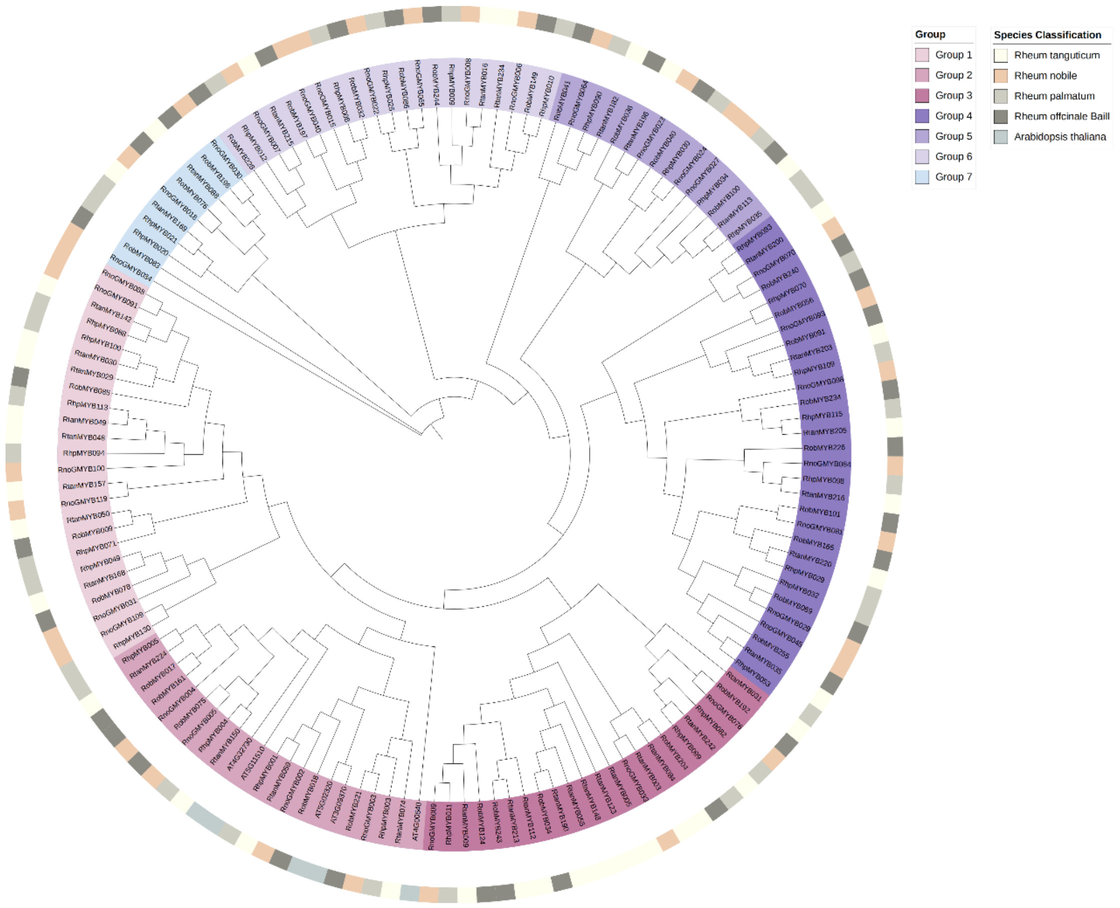

Fig. S5. The phylogenetic tree of MYB TFs subfamily classification system for four Rheum species and Arabidopsis (3R-MYB).

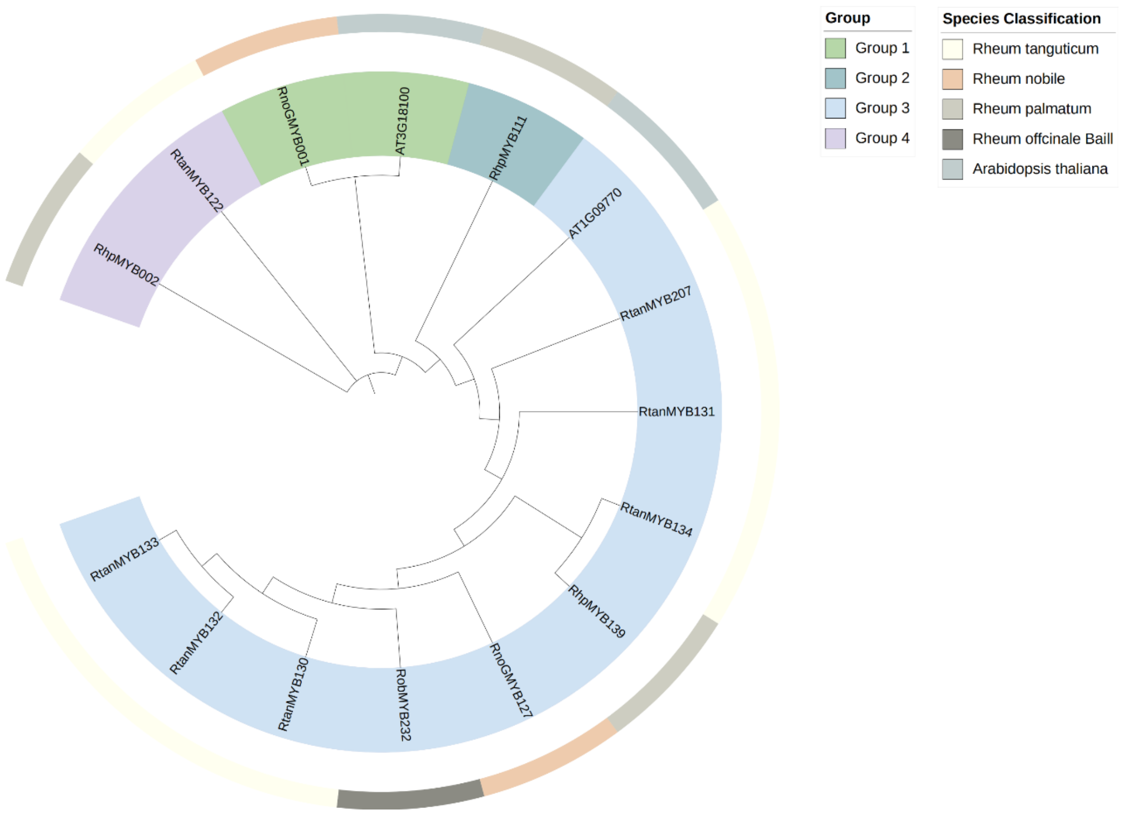

Fig. S6. The phylogenetic tree of MYB TFs subfamily classification system for four Rheum species and Arabidopsis (4R-MYB).

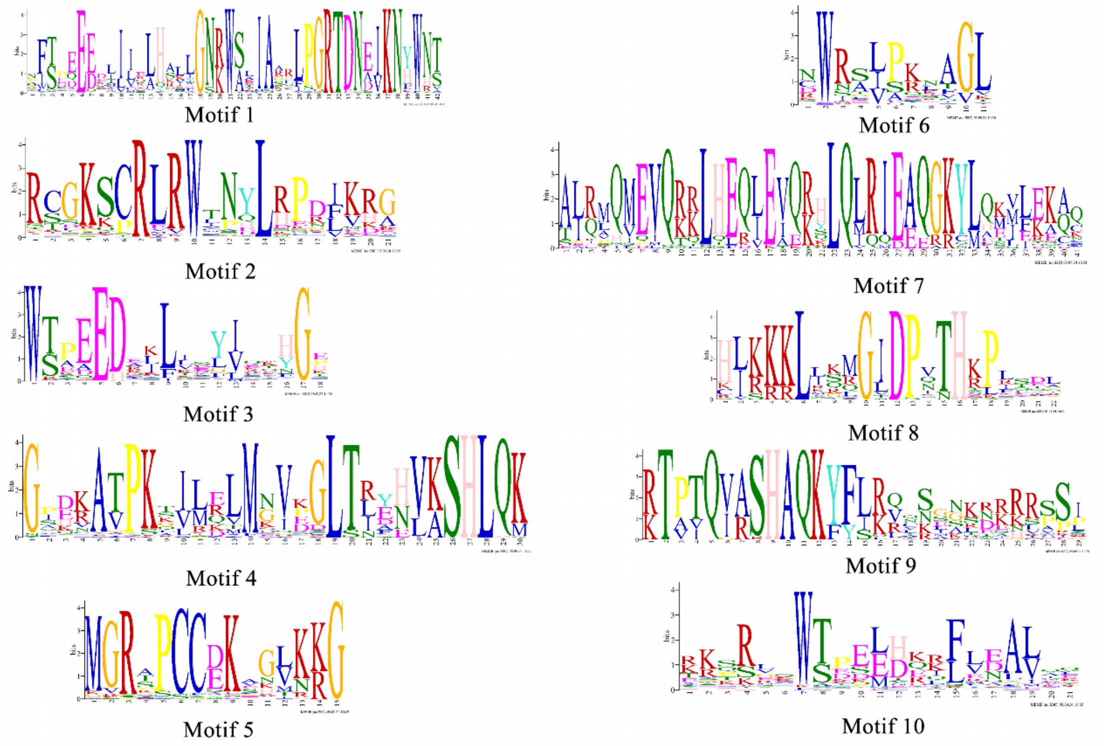

Fig. S7. The protein conserved motifs of MYB TFs in Rheum plants (*R. palmatum*).

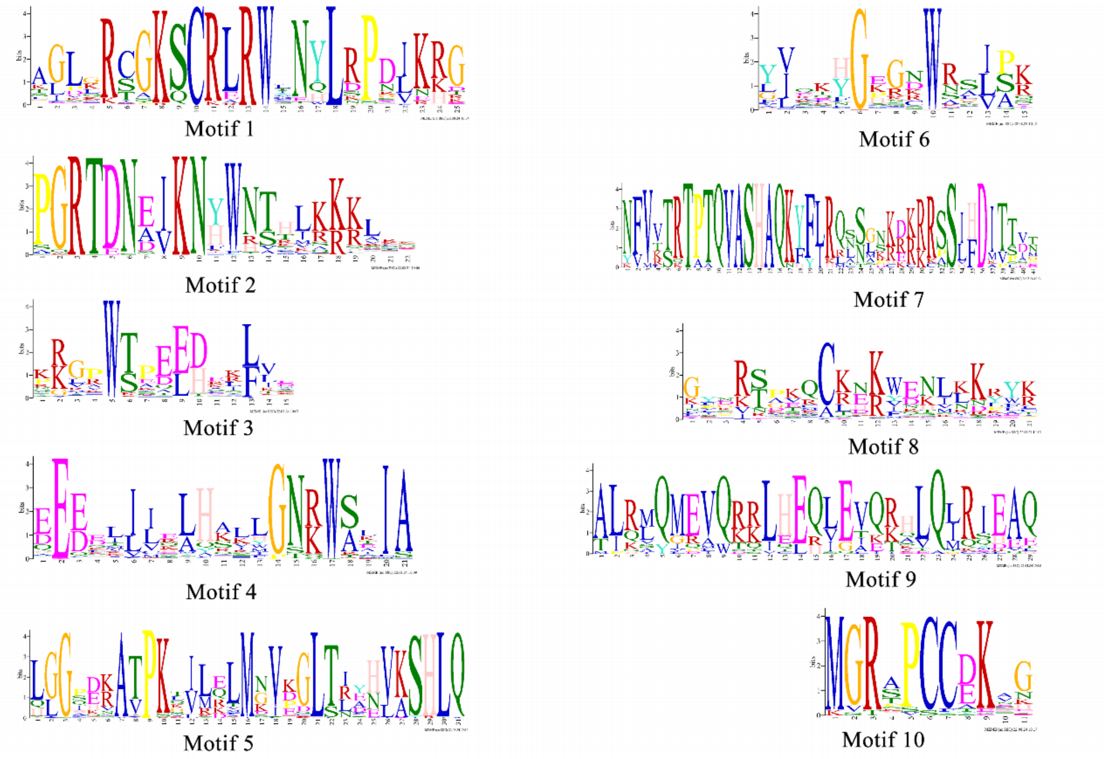

Fig. S8. The protein conserved motifs of MYB TFs in Rheum plants (*R. tanguticum*).

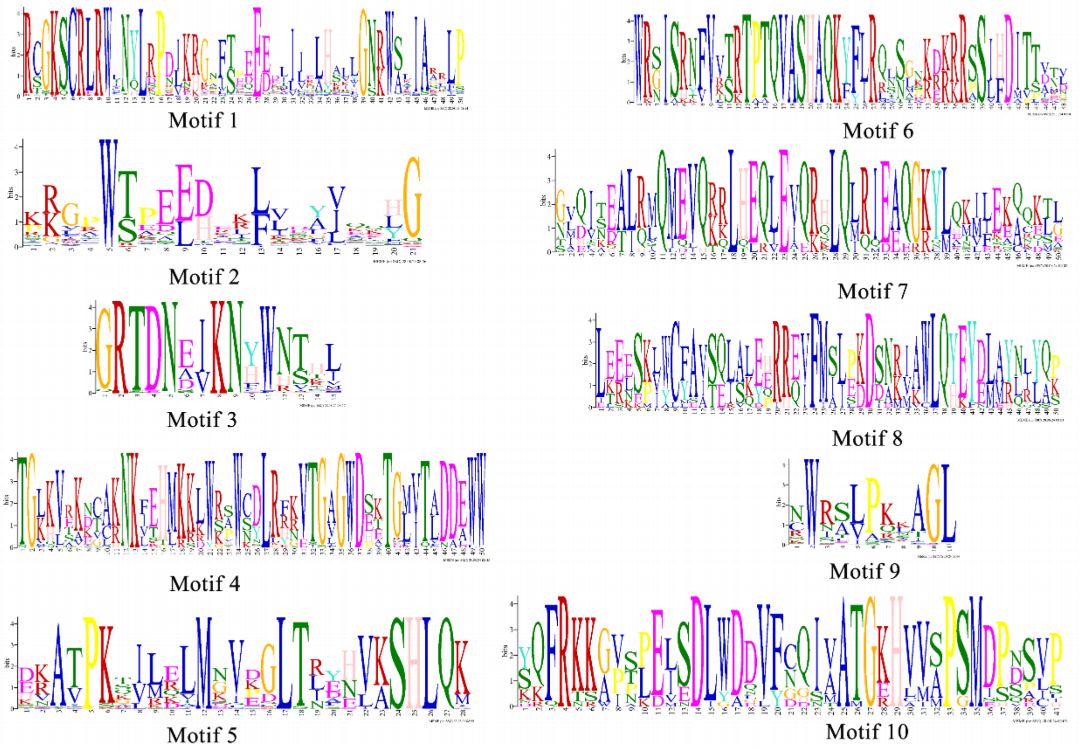

Fig. S9. The protein conserved motifs of MYB TFs in Rheum plants (*R. offcinale*).

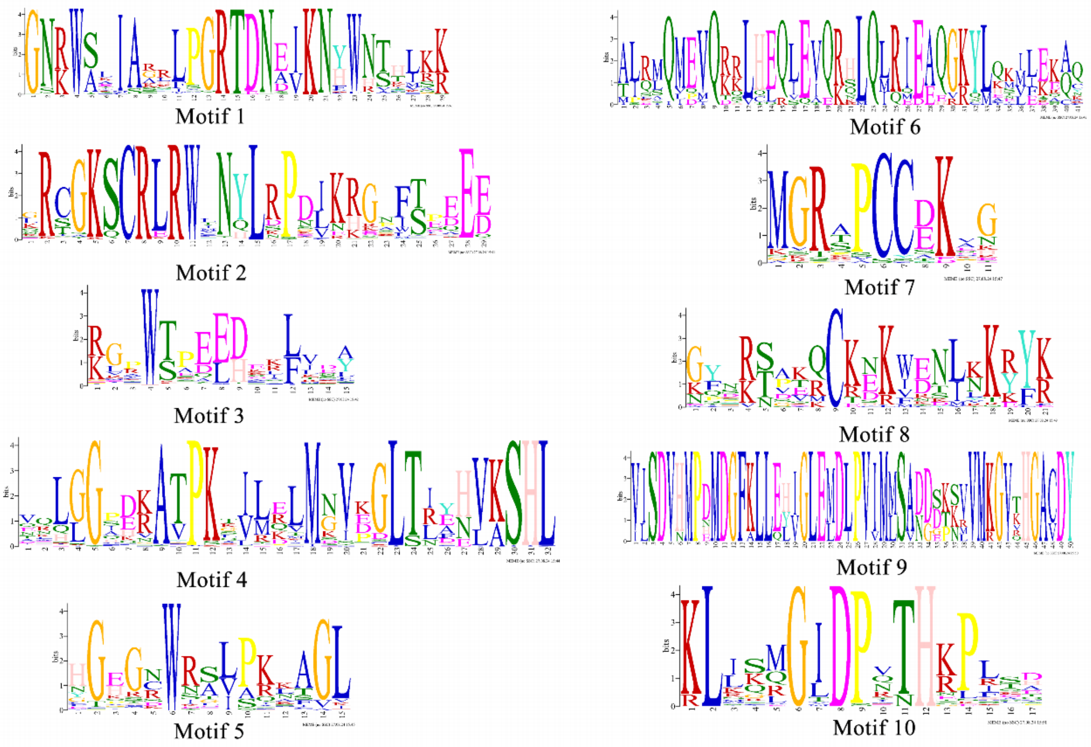

Fig. S10. The protein conserved motifs of MYB TFs in Rheum plants (*R. nobile*).

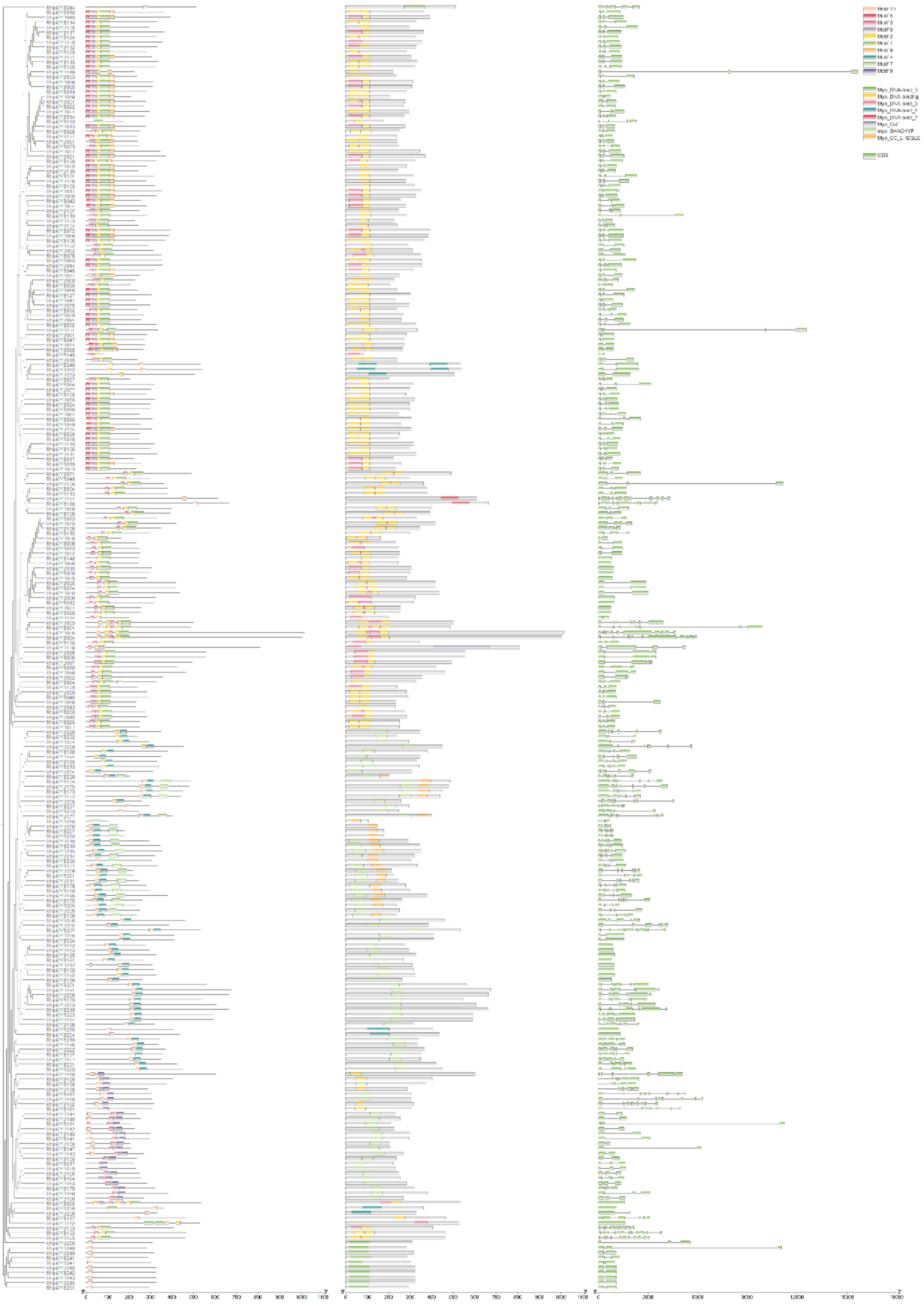

Fig. S11. Phylogenetic analysis, motif composition, conserved domains, and gene structure of MYB TFs in Rheum plants (*R. palmatum*).

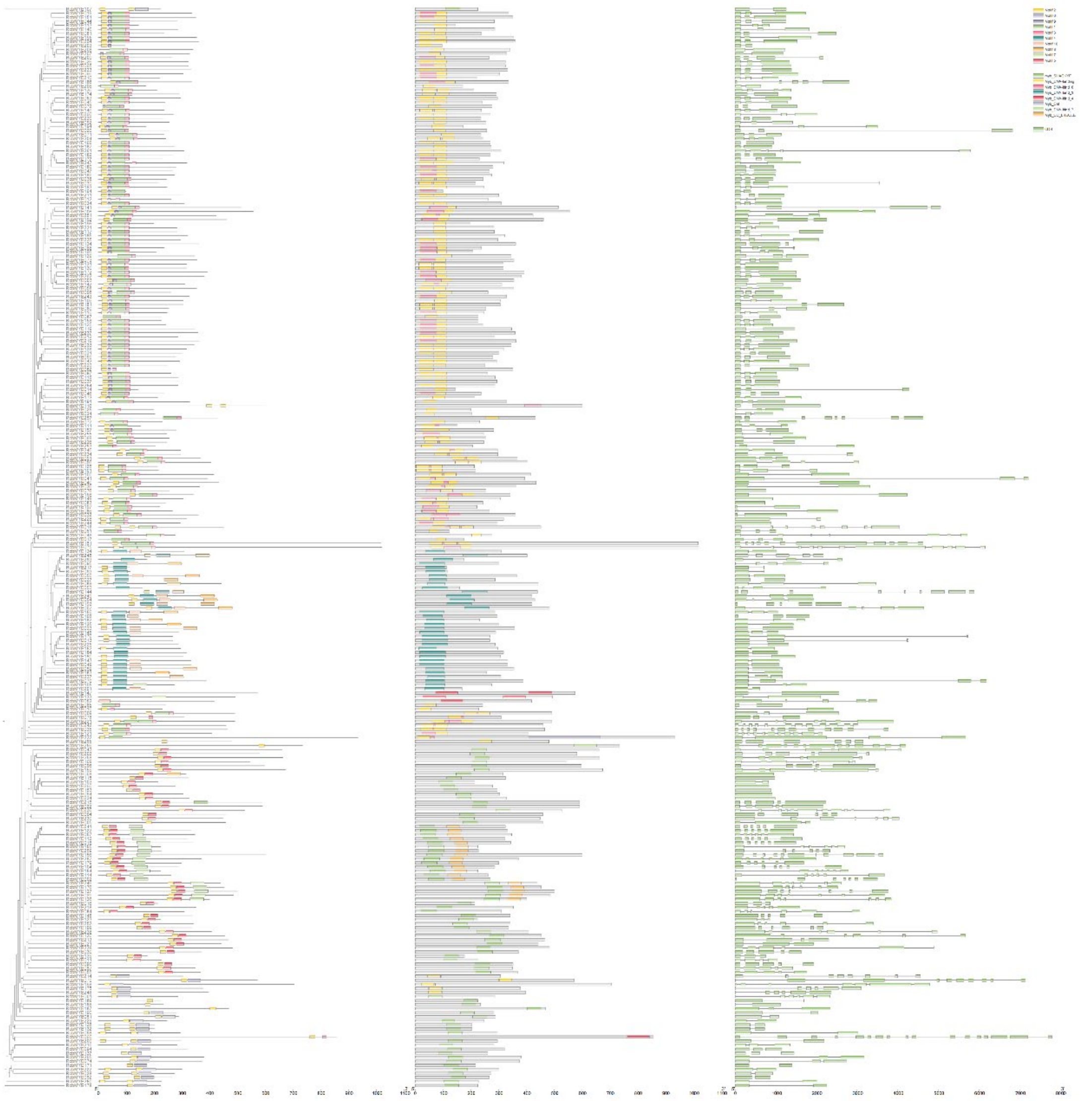

Fig. S12. Phylogenetic analysis, motif composition, conserved domains, and gene structure of MYB TFs in Rheum plants (*R. offcinale*).

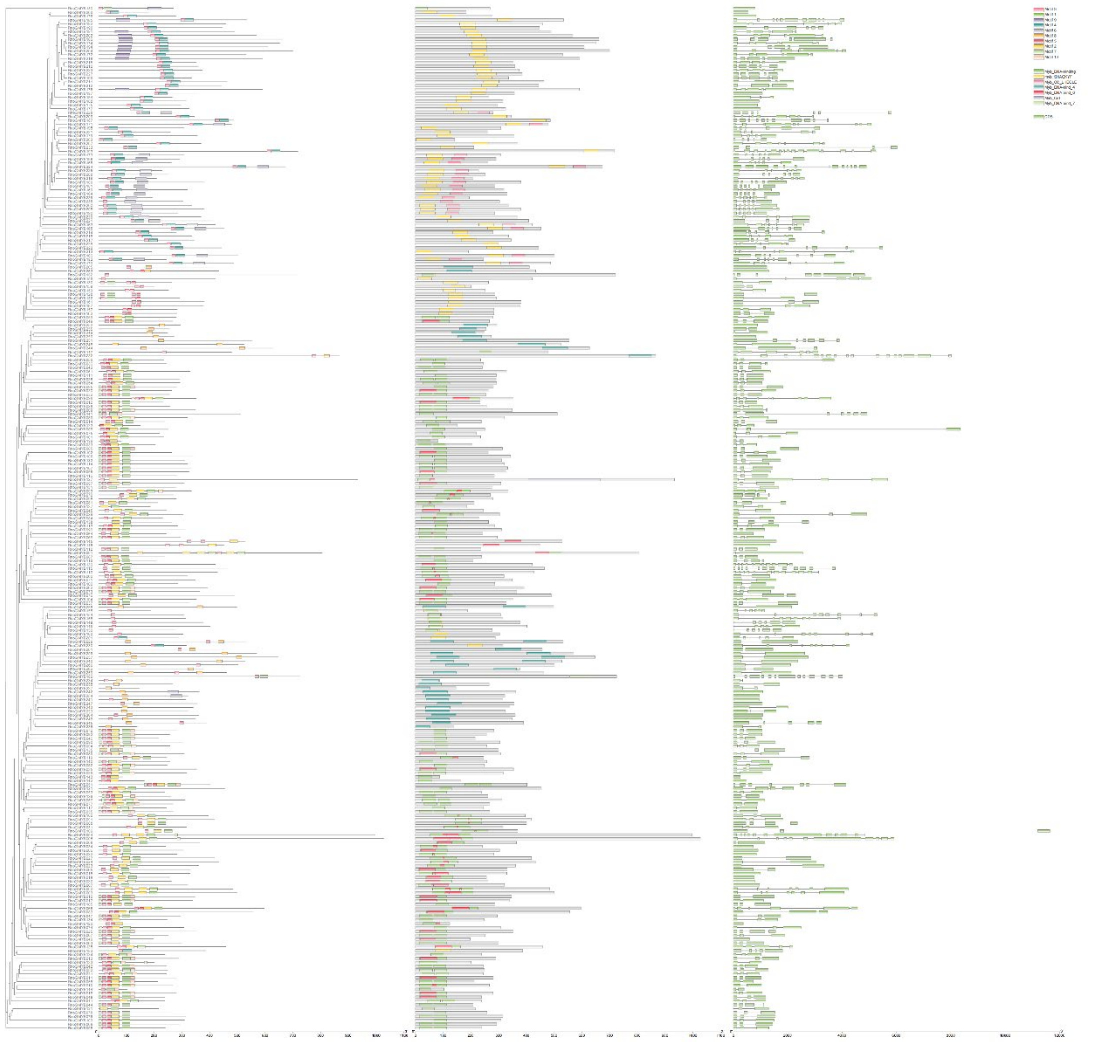

Fig. S13. Phylogenetic analysis, motif composition, conserved domains, and gene structure of MYB TFs in Rheum plants (*R. nobile*).

References

Zhao Shuo. Molecular regulatory mechanisms of anthraquinone biosynthesis in *Rheum tanguticum* explored by multi-Omics approaches [D]. University of Chinese Academy of Sciences, 2024. DOI: http://ir.nwipb.ac.cn/.
